# Existing evidence on the potential of soils constructed from mineral wastes to support biodiversity: a systematic map

**DOI:** 10.1186/s13750-024-00332-7

**Published:** 2024-04-08

**Authors:** Dakis-Yaoba Ouédraogo, Alix Lafitte, Romain Sordello, Florie Pozzi, Irina Mikajlo, José Hilario Rocha Araujo, Yorick Reyjol, Thomas Z Lerch

**Affiliations:** 1 PatriNat (OFB-MNHN-CNRS-IRD), 75005 Paris, France; 2 ECT, 77230 Villeneuve Sous Dammartin, France; 3 Institute of Ecology and Environmental Sciences of Paris (SU, CNRS, IRD, INRAE, UPEC, UPC), 32 Avenue Henri Varagnat, 93140 Bondy, France

**Keywords:** Anthroposol, Anthropogenic soil, Anthrosol, Artificial soil, Circular economy, Constructed Technosols, Construction and Demolition waste, Pedological engineering, Recycling

## Abstract

**Background:**

The development of cities and transport infrastructure produces a large volume of mineral waste (e.g. excavated earth material). At the same time, cities are increasingly trying to develop green infrastructures, given the ecosystem services they provide to people, but this comes with considerable economic and environmental costs associated with the transfer of fertile soil from rural areas to cities. In a circular economy approach, the reuse of mineral waste to build fertile soil is a substantial opportunity to reduce the economic and environmental costs of both mineral waste management and green infrastructure development. Soils constructed from these materials (constructed Technosols) must be able to support vegetation growth and become a suitable living environment for soil organisms. This requires ecological engineering to maximise the potential of constructed soils for biodiversity, both from a taxonomic and functional perspective. In this context, we systematically mapped the evidence related to the ability of soils constructed from mineral wastes to support biodiversity.

**Methods:**

We gathered published and grey literature through searches in two publications databases (Scopus and Web of Science Core Collection), one search engine (Google Scholar), nine organisational websites and through a call for literature. Titles, abstracts, and full-texts were successively screened using eligibility criteria. All included studies were described with coded variables and a database was produced. The extent of evidence was assessed and knowledge clusters and gaps were identified.

**Review findings:**

The searches yielded 9265 articles, and 153 articles were retained after the screening process. More than half of these articles were from European countries, with France leading the field with 40 articles, followed by Spain (15 articles) and Italy (10 articles). Most of the articles (75%) were produced after 2015. The main reasons for constructing soils from mineral waste were for mine rehabilitation (35%), waste recycling (16%) and experimental purpose (15%). The 153 articles were divided into 1962 studies, a study being a combination of a taxon, an intervention (i.e. soil construction) and a measured outcome. Among these studies, the most studied biological group is plants (69% of studies) and especially herbaceous species (32%), followed by microorganisms (17%) and invertebrates (14%). The most used type of mineral waste is mine waste (31% of studies) followed by excavated soil (16%) and demolition waste (14%). Finally, the most frequently measured outcome is plant growth (42% of studies), followed by organism abundance (16%) and diversity (10%).

**Conclusions:**

Three main knowledge clusters were identified which could be addressed in the future for full synthesis of the results: (1) How well do plants grow in soils constructed from mineral wastes? (2) What is the potential of soils constructed from mineral wastes to support biodiversity? and (3) How do microbial communities develop in soils constructed from mineral wastes? There is a lack of studies investigating several biological groups at the same time: only 6 articles out of 153 investigated the response of both plants, invertebrates and microorganisms to soil construction. More research is therefore needed on the ability to support a diversity of organisms.

**Supplementary Information:**

The online version contains supplementary material available at 10.1186/s13750-024-00332-7.

## Background

**Fig. 1:**
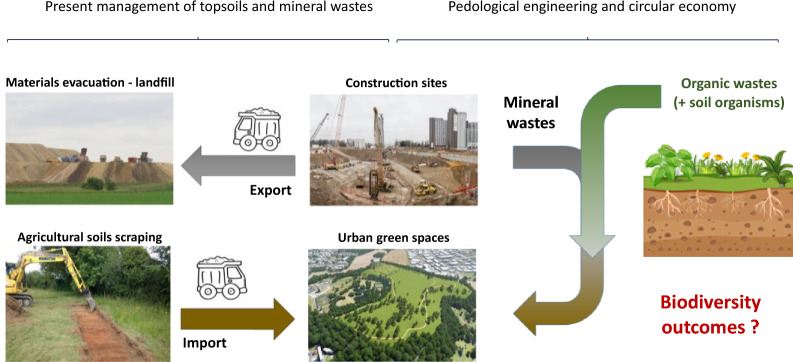
Diagram illustrating how the economic and environmental costs of the present management of mineral waste and topsoils could be reduced through pedological engineering in a circular economy approach (based on Freepik images and credit Le Parisien / Faustine Léo (landfill picture), L’opinion / Grandin de l’Epervier Jade (construction site picture), Pro24.fr (soil scraping picture) and ECT (green space picture)

In 2018, an estimated 55.3 percent of the world’s population lived in urban settlements. By 2030, urban areas are projected to house 60% of people globally and one in every three people will live in cities with at least half a million inhabitants [1]. The development of cities and transport infrastructure is accompanied by negative impacts as it is estimated that more than two billion of tons of solid wastes are produced every year [2]. A large proportion of these wastes are caused by construction and demolition activities (e.g. crushed concrete, pavements, excavated earth material, …). The management of excavated materials, considered as the most important waste, has substantial economic and environmental costs (e.g. greenhouse gas emissions), as they are most often stored in landfills outside the cities (Fig. 1). Although recycling practices are more and more encouraged by the development of innovative techniques [3], the amount of wastes and the distance to landfills both increase with urbanization.

At the same time, cities are increasingly trying to develop green infrastructures, given the ecosystem services they provide to people such as air filtration, micro-climate regulation, noise reduction, rainwater drainage, and recreational and cultural values [4]. To build these green infrastructures, natural soils are generally taken from surrounding rural areas, and this transfer also generates substantial economic and environmental costs (e.g. greenhouse gas emissions, loss of agricultural services, Fig. 1). This practice is also not sustainable because natural soil is a non-renewable resource given the very low rate of soil production (e.g. the rate of soil production has been measured to be between 0.01 and 59.4 mm per century, median 2.7 mm per century, [5]).

In a circular economy approach, the reuse of mineral wastes to build fertile soil is an opportunity to reduce the economic and environmental costs of both mineral waste management and green infrastructure development (Fig. 1). This approach, called pedological engineering, has emerged in the last 15 years. It consists of the deliberate mixture of wastes (organic and/or inorganic) and industrial by-products that are formulated and stacked in layers to create a new soil profile over in situ degraded substrates [6]. Soils constructed from these materials must be able to support vegetation growth and become a suitable living habitat for soil organisms [7, 8]. This requires both soil sciences knowledge and ecological engineering methods to maximise the potential of constructed soils for biodiversity, both from a taxonomic and functional perspective.

Soils constructed from mineral wastes can be classified according to the World Reference Base for Soil Resources [9] as *constructed Technosols*. Technosols “combine soils whose properties and pedogenesis are dominated by their technical origin. They contain a significant amount of artefacts (something in the soil recognizably made or strongly altered by humans or extracted from greater depths) or are sealed by technic hard material (hard material created by humans, having properties unlike natural rock) or contain a geomembrane. They include soils from waste (landfills, sludge, cinders, mine spoils and ashes), pavements with their underlying unconsolidated materials, soils with geomembranes and constructed soils”. While most Technosols are unintentionally inherited from human activities (e.g. urbanisation), *constructed Technosols* are created from parent materials selected and mixed intentionally for the purpose of assay [10]. They can therefore offer an alternative to the exportation of waste from cities but also the importation of topsoil from peri-urban areas. So far, the potential of constructed Technosols to support biodiversity has not been well documented.

A mapping of the articles mentioning the keyword "Technosols" in their title, abstract or keywords has been published recently [11] and presented the fields of use of Technosols, which include mining and industrial activity, urban areas, investigation of Technosol pedogenesis, classification, formulation and analytical methods in general, and diverse activities such as agriculture, recreational activities, natural spaces, waste landfill and aquaculture. The use of constructed soils made from organic and inorganic wastes for restoration of mined lands, urban or industrial areas, or for urban greening has also been recently reviewed [12–14]. The different waste materials used, the outcomes assessed, as well as the issues related to the use of constructed soils were described. Except the review by Deeb et al. [12] focusing on green infrastructure, none of these reviews mention the method used to carry out the synthesis, so they are not reproducible and the risk of bias due to the selection of particular studies cannot be assessed. They also did not specifically examine how biodiversity issues were taken into account in Technosols construction, which is an important question given that the construction of Technosols is likely to increase. In this paper, we therefore systematically mapped the evidence related to the potential of soils constructed from mineral wastes to support biodiversity.

### Topic identification and stakeholder input

The specific issue addressed in this work was clarified during discussions with members of the ECT company that funded this systematic map. This company specialised in the storage of inert materials from building and public works sites and manages the storage of approximately 15 million tonnes of excavated soil per year in the Île-de-France region.

## Objective of the review

### Primary question

What evidence exists on the potential of soils constructed from mineral wastes to support biodiversity?

### Components of the primary question

Population: All living organisms (flora, fauna, microbiota, fungi, etc.).

Intervention: Construction of soil from mineral wastes (e.g. excavated materials or sediment, concrete blocks, decontaminated soils).

Comparator: Other soils or other constructed soils; before adding the soil constructed from mineral wastes.

Outcomes: All outcomes related to living organisms (presence, abundance, diversity, activity, etc.) and biological processes.

## Methods

The systematic map followed the Collaboration for Environmental Evidence Guidelines and Standards for Evidence Synthesis in Environmental Management [15] and conforms to reporting standards for systematic evidence synthesis (ROSES [16], see Additional file 1). The systematic map is based on an a priori protocol registered in PROCEED, the global database of prospectively registered evidence reviews and syntheses in the environmental sector [17].

### Deviations from the protocol

Some deviations from the protocol occurred during the conduct of the review process to improve the robustness of the method. First, the question was slightly rephrased from “What evidence exists on the potential of Technosols constructed from mineral wastes to host biodiversity?” to “What evidence exists on the potential of soils constructed from mineral wastes to support biodiversity?” and the components of the question were also slightly rephrased accordingly. Second, another organisational website was identified and searched during the literature search, and a call for literature among stakeholders was performed. Third, the eligibility criteria were clarified and narrowed after discussing the disagreements that arose during the screening consistency checks. Fourth, the studies were not coded by a single trained reviewer but by two reviewers who checked the consistency of their coding beforehand. Finally, two additional variables were coded from the studies: the description of the control and whether the data needed for a quantitative or a narrative synthesis were present.

### Search for articles

#### Search terms and strings

A search string combining keywords describing the intervention element of the question (soils constructed from mineral wastes) was built through a scoping exercise in the Web Of Science Core Collection (WOS CC) database (Additional file 2). The search string that gave the highest comprehensiveness and specificity is as follows (Web Of Science format):

TS = (technosol$ OR technosoil$ OR techno-soil$ OR anthroposol$ OR anthroposoil$ OR "anthropogenic soil$" OR anthrosol$ OR anthrosoil$ OR "construct* soil$" OR "engineered soil$" OR "rebuilt soil$" OR "artificial soil$" OR "fabricated soil$" OR "structural soil$" OR "excavated soil$" OR "inert soil$" OR "excavated material$" OR "excavated earth" OR "inert material$" OR "surplus soil$" OR "urban construction waste$").

#### Search limitations

The search was conducted without date limitations, using terms in the English language, but many of the organisational websites were searched in French.

#### Search sources

The main search was conducted on June 17th 2022 on two bibliographic databases: Scopus (Elsevier) and Web of Science Core Collections (Clarivates Analytics). On Scopus, the search string was adapted to fit the required format as follows:

TITLE-ABS-KEY(technosol OR technosoil OR techno-soil OR anthroposol OR anthroposoil OR "anthropogenic soil" OR anthrosol OR anthrosoil OR "construct* soil" OR "engineered soil" OR "rebuilt soil" OR "artificial soil" OR "fabricated soil" OR "structural soil" OR "excavated soil" OR "inert soil" OR "excavated material" OR "excavated earth" OR "inert material" OR "surplus soil" OR "urban construction waste").

Access to the databases was through a CNRS (the French National Centre for Scientific Research) subscription, allowing access to the following WOS CC Citation Indexes: Science Citation Index Expanded (SCI-EXPANDED, 1900-present); Social Sciences Citation Index (SSCI, 1956-present); Arts & Humanities Citation Index (A&HCI, 1975-present); Conference Proceedings Citation Index- Science (CPCI-S, 1998-present); Conference Proceedings Citation Index- Social Science & Humanities (CPCI-SSH, 1998-present); Emerging Sources Citation Index (ESCI, 2015-present); Current Chemical Reactions (CCR-EXPANDED, 1985-present, includes Institut National de la Propriété Industrielle structure data back to 1840); Index Chemicus (IC, 1993-present).

Additional search was performed on June 17th 2022 using Google Scholar web search engine. Searches were performed on the title using the search string:

technosol OR technosols OR technosoil OR technosoils OR anthroposol OR anthroposols OR anthroposoil OR anthroposoils OR anthrosol OR anthrosols OR anthrosoil OR anthrosoils.

All records were kept and results were extracted using the software Publish or Perish (version 7.15.2643.7260, https://harzing.com/resources/publish-or-perish, version accessed 16 March 2020).

Additional searches were also performed on October 3rd—7th 2022 on the following nine organisational websites (Additional file 3):

- Food and Agriculture Organization (FAO) (https://www.fao.org/about/en/)
- European Circular Economy Stakeholder Platform (https://circulareconomy.europa.eu/platform/en)
- French Agency for Ecological Transition (ADEME) (https://www.ademe.fr/)
- French Biodiversity Agency (OFB) (https://www.ofb.gouv.fr)
- Resources centre for ecological engineering of the French Biodiversity Agency (https://www.genieecologique.fr/)
- Paris Region Institute (https://www.institutparisregion.fr/)
- French Geological Survey (BRGM) (https://www.brgm.fr/)
- Centre for landscape and urban horticulture (Plante & Cité) (https://www.plante-et-cite.fr/)
- French Centre for Studies and Expertise on Risks, Environment, Mobility and Urban planning (Cerema) (https://www.cerema.fr/fr)

#### Supplementary searches

A call for literature at the International Conference-Exhibition on Soils, Sediments and Water (Intersol 2022) was done on 21–23 June 2022 through flyers and a poster. Some articles were also sent spontaneously by colleagues during the conduct of the review.

#### Estimating the comprehensiveness of the search

To assess the comprehensiveness of the search, a list of 20 articles answering the review question was built, of which 19 were indexed in WOS CC (Additional file 2). These articles were mainly identified from the references of reviews on the subject [12–14] and a master thesis aiming at reviewing all the literature on Technosols [18]. Our search string was able to retrieve all 19 articles indexed in WOS CC (Additional file 2), and the article that was not indexed (a PhD thesis) was retrieved by the search on Google scholar.

#### Search results

Search results from publication databases and Google Scholar were combined and deduplicated in CADIMA [19]. Other searches were added and deduplicated manually in a Microsoft Excel spreadsheet before full-text screening.

### Article screening and study eligibility criteria

#### Screening process

Articles were screened for eligibility in two successive stages. First, search results from publication databases and Google scholar were screened on titles and abstracts using CADIMA [19]﻿. The included articles were then exported in a Microsoft Excel spreadsheet to be screened on full text. Articles retrieved from other searches were added at this stage. Articles without an abstract and retained based on title screening were directly screened on their full-text. Articles with unclear eligibility status during title/abstract screening were included for full-text screening. The list of articles with unclear eligibility status or excluded after full-text screening is provided with reasons for unclear eligibility or exclusion (Additional file 4).

Screening was performed by two reviewers (DYO, AL). Before the screening, the consistency between their decisions was assessed by computing Randolph’s Kappa coefficient on a random sample of articles (600/9265, 6.5% for title/abstract, and 90/1028, 8.8% for full-text). During all screening processes and consistency checks, reviewers never had to screen their own articles. A topic expert (FP) took part in the consistency checks to compare the reviewer's decisions with those of an expert and to inform discussions on the clarification of eligibility criteria. Consistency checks were performed in two steps, the three reviewers discussing all disagreements after each step: first on 100 (Kappa = 0.67) then on 500 titles/abstracts (Kappa = 0.82), and first on 30 (Kappa = 0.73) then on 60 full-texts (Kappa = 0.69). The kappa values obtained (> 0.6) were considered an acceptable level of agreement.

#### Eligibility criteria

Articles were screened according to the eligibility criteria described in Table 1. The criteria Language, Type of document, and Type of content were assessed exclusively at full-text screening.

**Table 1:** Eligibility criteria

Include	Exclude
*Population* - all living organisms (flora, fauna, microbiota, fungi, etc.)	*Population*
*Intervention* - construction of soil from mineral wastes (e.g. excavated materials or sediment, concrete blocks, decontaminated soils, mudflow, rubble). The "waste" nature of the materials used to construct the soil must be mentioned or understood with the context. The soil can be built by mixing all the materials or by stacking them in layers - soil construction using only one material that is a mineral waste - construction of soil from mineral wastes to cover a polluted area/soil - soil spray made with mineral waste	*Intervention* - Technosols (e.g. urban soils) that are not constructed - construction of soil but not from mineral waste - construction of soil using materials explicitly stated as polluted/toxic. If the study tests for toxicity, accumulation of toxic elements, or refers to the potential toxicity of the materials in context, they are considered toxic
*Comparator* - other soils or other constructed soils, or before adding the soil constructed from mineral wastes	*Comparator* - No comparator
*Outcome* all outcomes related to living organisms (presence, abundance, diversity, activity, etc.) and biological processes (e.g. respiration, denitrification or carbon mineralization resulting from microbial activity)	*Outcome* - Soil physico-chemical properties or fertility, hydrological properties - tissue content, accumulation or uptake in mineral elements, metals or pollutants
*Language* - English and French	*Language*
*Type of document* - journal article, report, book chapter, conference proceeding article, Ph.D. or M.Sc. thesis, preprint	*Type of document* - presentations, editorial materials, news, abstracts, posters, and datasets
*Type of content* - in-situ or ex-situ studies	*Type of content* - reviews, meta-analyses, modelling studies without experimental data, discussion or opinion papers

To assess the extent to which evidence is missing because articles were excluded for language reasons, articles excluded because of language (n = 76) were retrospectively screened for eligibility. Three reviewers understanding Portuguese (JHRA), Czech, Polish, Ukrainian and Russian (IM), and German (TZL) screened articles written in these languages. For other languages (Chinese, Finnish, Indonesian, Italian, Japanese, Korean, Lithuanian, Spanish), Deepl or Google Translator was used.

#### Study validity assessment

No critical appraisal of the studies has been performed for the systematic map.

#### Data coding strategy

Articles included after the screening process were split into studies, a study being a combination of a taxon, an intervention and an outcome, and the following variables were extracted in a spreadsheet (Additional file 5, sheet "coding book"):

- Bibliographic information (unique identifier, source, title, authors, journal, year, DOI, language and type of document)
- General description of the study (country, location, experimental system, reason for intervention, land use for in situ studies)
- Description of the population (taxon, and if the population is a construction element of the soil)
- Description of the intervention (mineral waste(s) used, other materials used including living organisms, organisation of materials, age of the constructed Technosol/time since intervention, and type of comparator)
- Description of the outcome and information whether the article includes the data needed for a quantitative or a narrative synthesis.

The coding was distributed among two reviewers (DYO, RS), that never had to code their own articles. Before the actual coding, a random sample of studies (215/1962, 10.9%) was coded independently by both reviewers, all disagreements were discussed and the coding book was clarified where necessary. During coding, the missing or unclear information was coded as such.

#### Data mapping method

A database (Microsoft Excel sheet) of all included studies and their coded data was produced (Additional file 5). The evidence was first mapped at the article level for the source, document type, reason for intervention, geographical and chronological distribution of evidence. Using the list of the taxa studied (i.e. with an outcome measured) in each article we also assessed how many different biological groups (plants and/or invertebrates and/or microorganisms) were investigated by the article. We also analysed the co-occurrence of article keywords using the VOSviewer software version 1.6.19 [20] to identify how keywords are used in the literature to describe the question of our systematic map. The keywords defined by the authors and with a minimum of three occurrences were retained for the analysis. A thesaurus file was used to merge keywords in singular and plural form or with/without a hyphen, etc.

Second, the evidence was mapped at the study level, describing study characteristics and frequency distribution. A heatmap of the frequency of the studies into the types of population and types of outcome studied was produced, and knowledge gaps and clusters were identified according to the number of studies and relevance to stakeholders.

## Review findings

### Review descriptive statistics

**Fig. 2:**
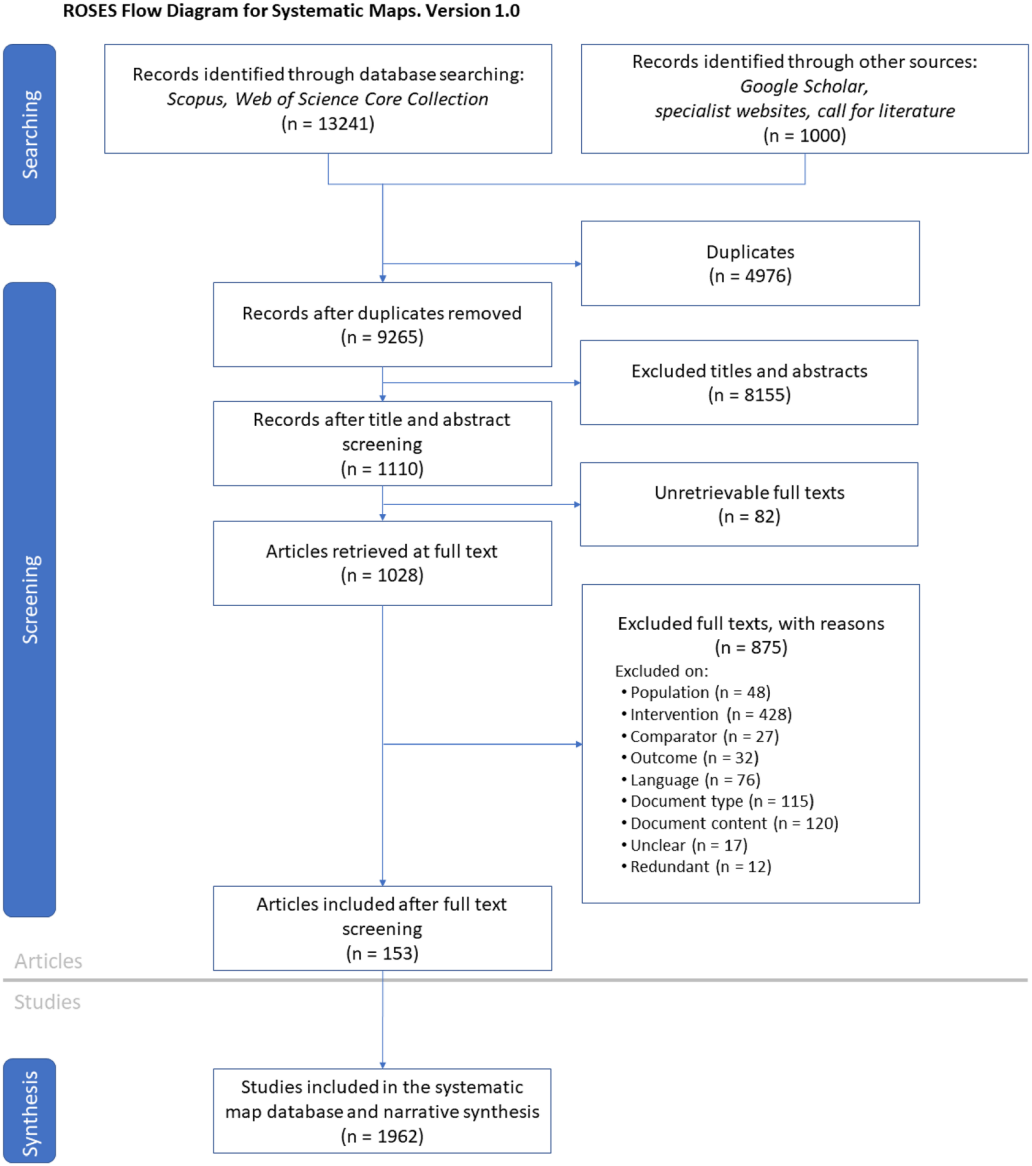
ROSES flow diagram [21] of the systematic map

We retrieved 7529 and 5712 records from the searches in the Scopus and Web Of Science Core Collection databases, respectively. The search in Google Scholar gave 979 records and searches on organisational websites 16 records. One article was sent following our call for literature, and 4 were sent spontaneously by colleagues. This resulted in 14,241 records reduced to 9265 records after duplicate removal (Fig. 2). Among them, 1110 remained after title/abstract screening and we could not retrieve 82 full texts (7.4%) resulting in 1028 full texts to screen. A total of 875 articles were excluded at full-text screening, mainly because of irrelevant intervention (38.6%), irrelevant document content (10.8%) or type (10.4%). All articles excluded, with unclear status, or for which we could not obtain the full text are listed in Additional file 4. In the end, 153 articles answer the review question, corresponding to 1962 studies, a study being a combination of a taxon, an intervention and an outcome, one article often comprising several studies.

### Description of the articles

Among the 153 articles answering the review question, 123 were retrieved from publication databases (80.4%), 24 from Google Scholar (GS, 15.7%), 3 from the website of the Centre for landscape and urban horticulture (Plante & Cité), 1 from the call of literature and 2 were spontaneously sent by colleagues. A substantial number of articles were written in French (13.1%), the others being written in English (86.9%). Of the 76 articles excluded on the language criterion and screened a posteriori, three appeared to answer the review question (one in Chinese, one in German and one in Portuguese, Additional file 4), which highlights the limitations of not considering all languages in evidence synthesis.

**Fig. 3:**
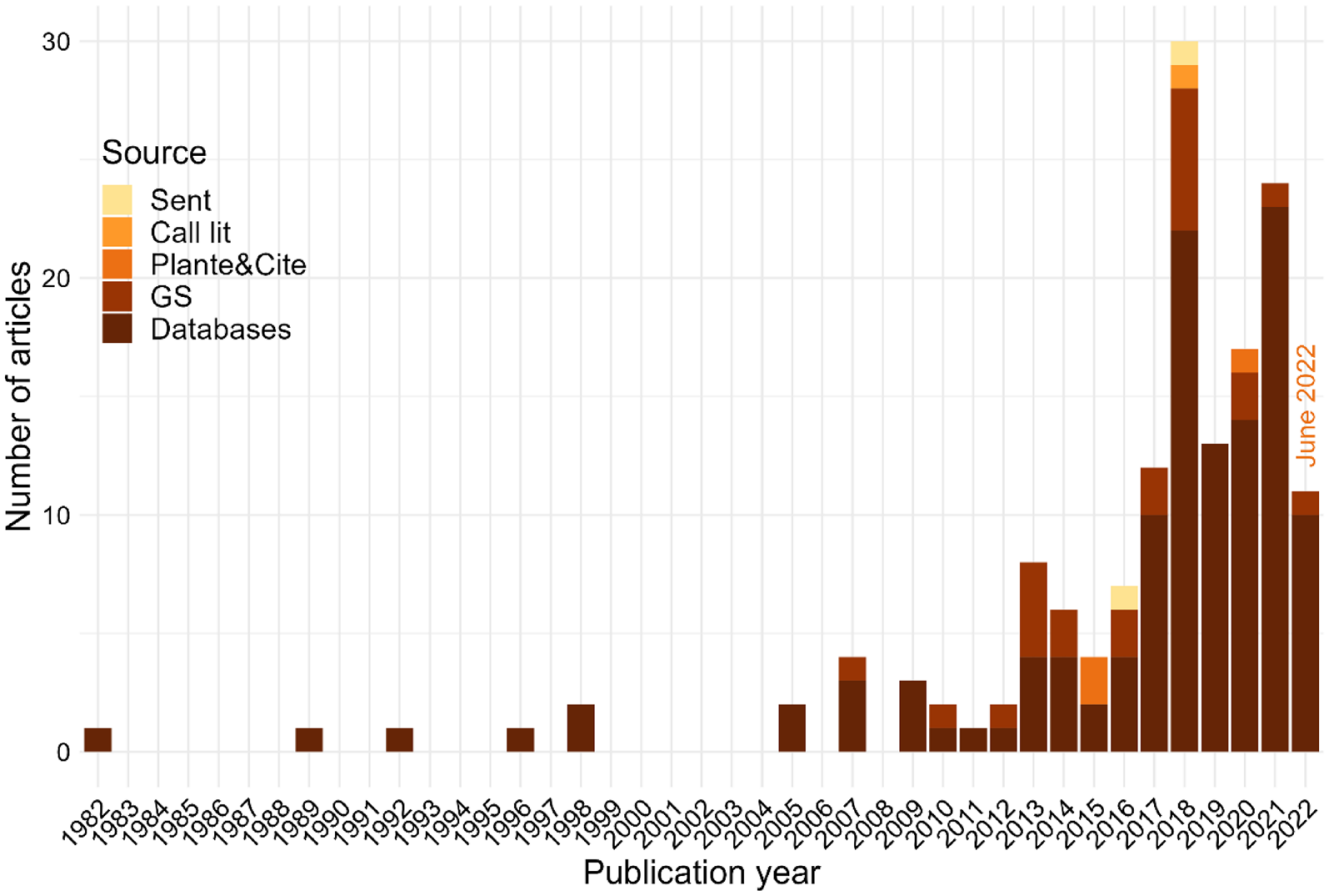
Chronological distribution of the articles until June 2022, with information on their sources (Databases are Web of Science Core Collection and Scopus)

Articles are mostly journal articles (82.4%), then PhD theses (9.8%), conference proceedings articles (3.9%), reports (2.6%) and MSc theses (1.3%). The oldest article was published in 1982 and more than two-thirds of the articles have only been produced since 2015 (Fig. 3).

**Fig. 4:**
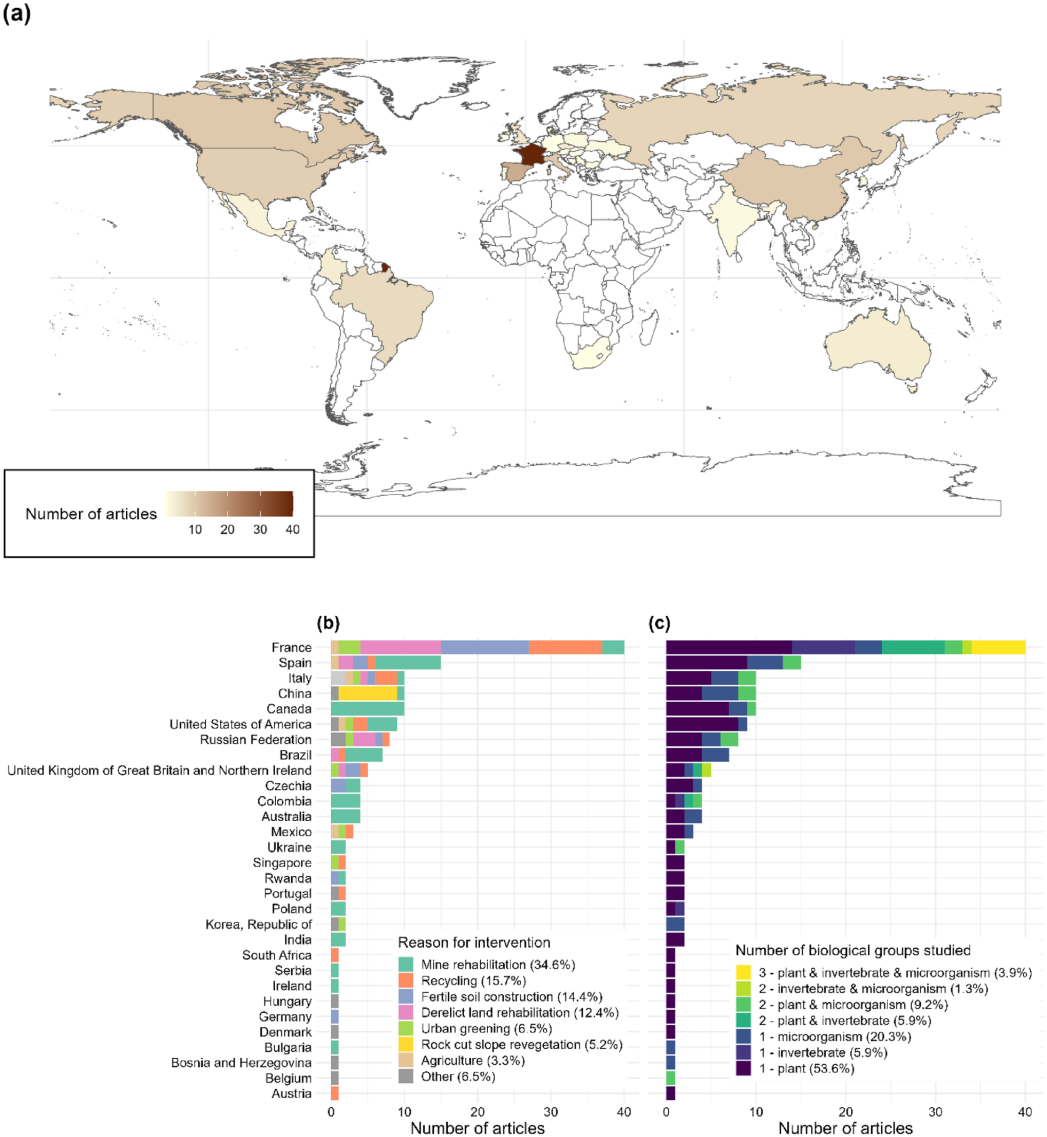
Geographical distribution of the articles (**a**) with information on the reasons for intervention (**b**) and on the number of biological groups that were studied (**c**)

The leading country is France with 40 articles (26.1%), then Spain (15 articles, 9.8%) and Italy, China and Canada with 10 articles each (6.5%, Fig. 4). When considering only journal articles written in English and retrieved from publication databases to remove the bias due to supplementary searches being focused on French literature, we found the same pattern of European countries leading the field with France (20 articles) then Spain (14 articles), Italy (10 articles), China (9 articles) and Canada (8 articles).

The main reasons for constructing a Technosol were for mine rehabilitation (34.6%), recycling (15.7%), experimental construction of fertile soil (14.4%), and derelict land rehabilitation (12.4%, Fig. 4b). Some countries showed very specific reasons for intervention. For example, Technosol construction in Canada, Colombia or Australia was exclusively for mine rehabilitation. The construction of Technosol for rock cut slope revegetation was exclusively performed in China, using the particular technic of soil spraying.

Most articles measured the response on a single biological group (plants 53.6%, microorganisms 20.3% or invertebrates 5.9%) and very few addressed the three groups together (3.9%), all located in France (Fig. 4c).

**Fig. 5:**
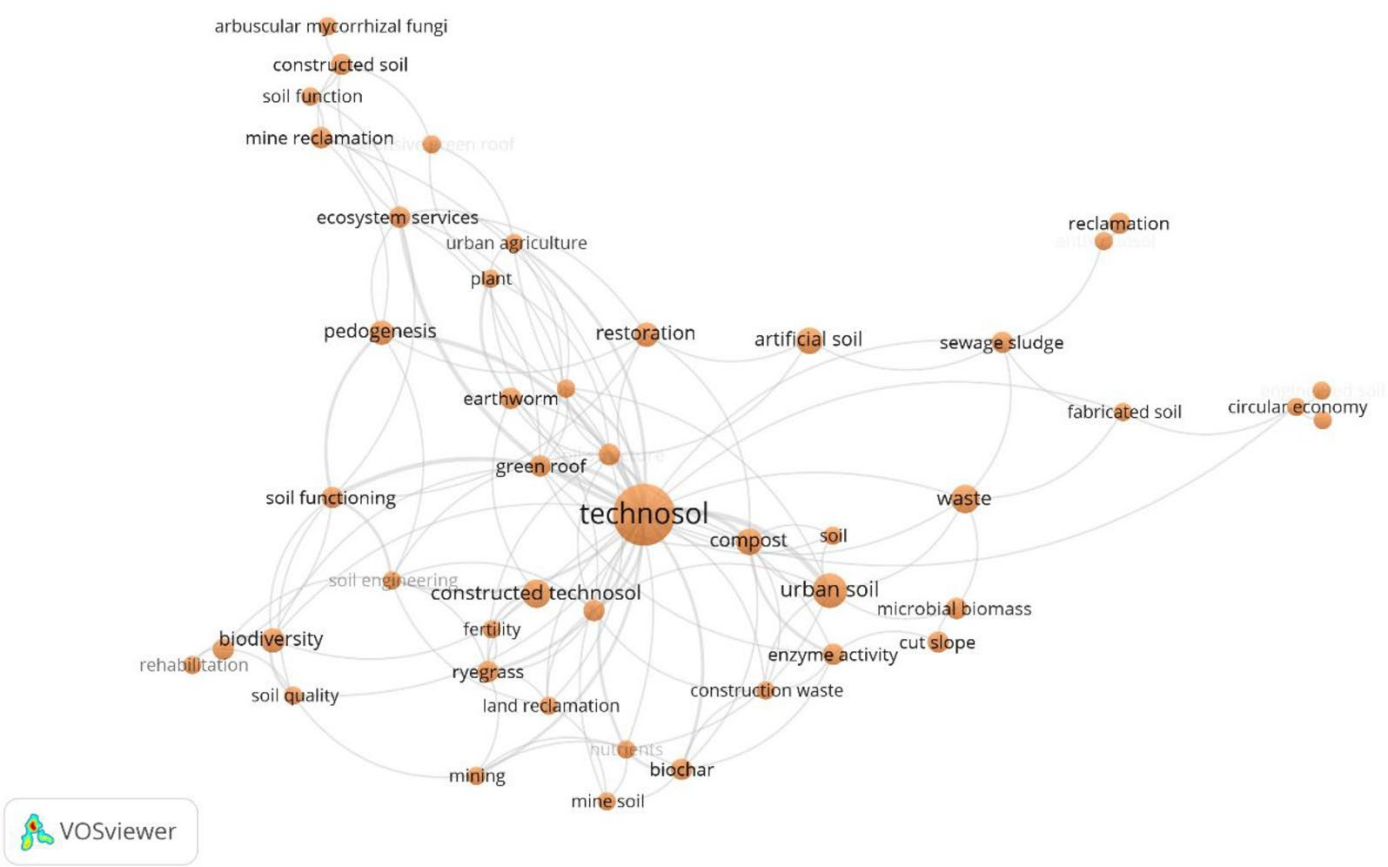
Network of the keywords defined by the authors of articles about the potential of soils constructed from mineral wastes to support biodiversity. From the 153 articles included in the systematic map, only the 138 that had defined keywords were analysed. Only keywords with a minimum of three occurrences are presented here. The bigger the circle, the more frequently the keyword appears in the literature. The lines connect the keywords that appear together

The analysis of the co-occurrence of authors keywords revealed that 46 keywords co-occurred at least three times in the literature (Fig. 5). The keywords used were mainly to describe the constructed Technosols (e.g. fabricated or artificial soil, urban soil, anthroposol, engineered soil), the wastes (e.g. construction waste, compost, sewage sludge), the usage of the constructed Technosols (e.g. reclamation, urban agriculture, green roof), and biodiversity. The keywords used to describe biodiversity were biodiversity, arbuscular mycorrhizal fungi, plant, earthworm, ryegrass, microbial biomass and enzyme activity.

### Description of the studies

#### Taxa and outcome studied

**Fig. 6:**
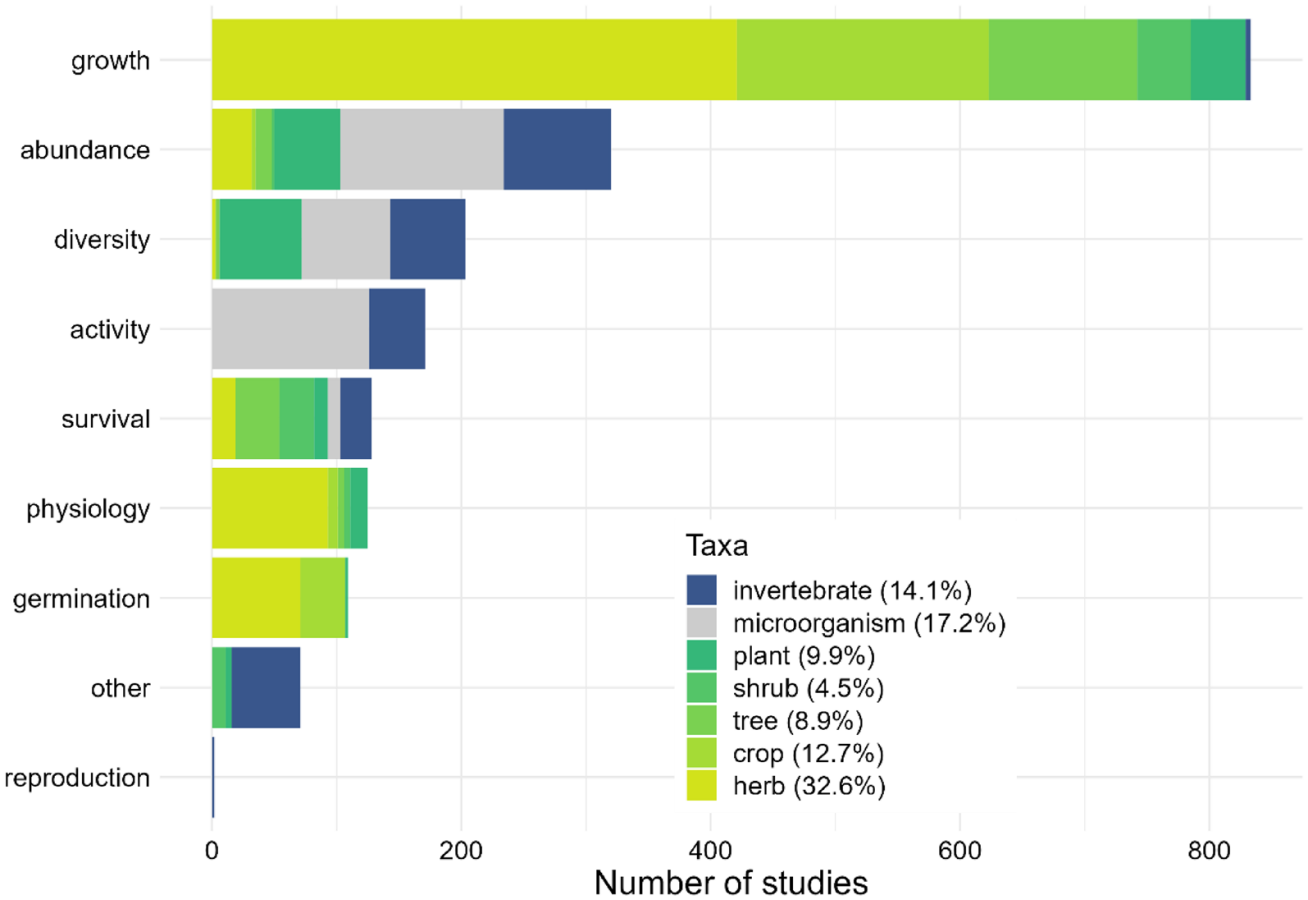
Distribution of the 1962 studies by taxa and outcome measured

The far most studied biological group is plants (68.7% of the studies), especially herbaceous plants (32.6%), followed by microorganisms (17.2%) and invertebrates (14.1%, Fig. 6). The far most studied outcome is growth (42.5% of the studies), especially plant growth (42.3%), followed by abundance (16.3%) and diversity (10.3%) of organisms.

#### Intervention

**Fig. 7:**
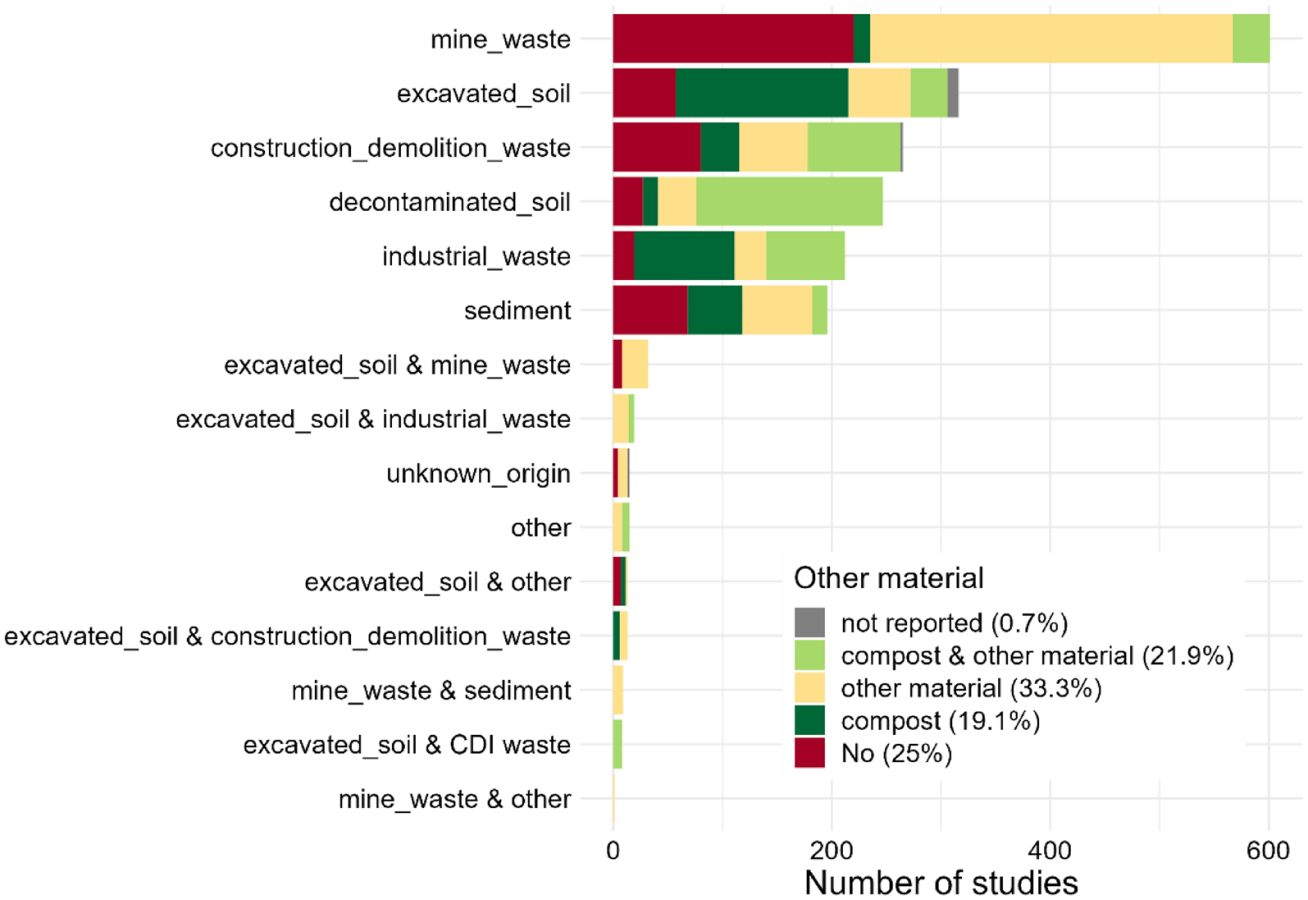
Distribution of the 1962 studies by type of mineral waste

The most used waste materials for Technosol construction are mine waste (e.g. overburden, spoil, stockpiled topsoil, 30.6% of the studies), excavated soil (16.1%), construction and demolition waste (e.g. concrete, rubble, 13.5%), decontaminated soil (12.6%), industrial waste (e.g. bricks, ballast, tiles, 10.8%) and sediment (10%, Fig. 7). Constructed Technosols were made of mineral waste alone (25% of the studies) or in combination with other materials (e.g. sludges, ashes, natural soils, 33.3%) or compost (19.1%). Most studies assessed biodiversity between 1 and 12 months after the intervention, but a certain number of studies (19.9%) also assessed biodiversity in the longer term (between 2 and 5 years, Table 2).

**Table 2:** Time after intervention

Time after intervention	Number of studies	%
≤ 1 month	205	10.4
> 1 month & ≤ 12 months	914	46.6
> 1 year & ≤ 2 years	119	6.1
> 2 years & ≤ 5 years	391	19.9
> 5 years & ≤ 10 years	109	5.6
> 10 years	162	8.3
Not reported	62	3.2

#### Comparator

For 29.4% of the studies the biodiversity in the constructed Technosols can be compared to that of a reference soil (natural soil or standard plantation medium). When this was not possible, the comparison recorded was, in order of preference (cf. Additional file 5, sheet "coding book"), before the intervention (0.6% of the studies), with another Technosol constructed from mineral waste (68.5%), with a constructed Technosol but not from mineral waste (0.8%), and with a polluted soil (0.8%).

### Knowledge clusters

#### How well do plants grow in Technosols constructed from mineral wastes?

A first knowledge cluster of 829 studies addresses the most measured outcome for the most studied biological group: plant growth (Table 3). This cluster is reduced to 744 studies with data for quantitative and/or narrative synthesis, and 193 studies comparing plant growth between a Technosol constructed from mineral waste and a reference soil. These studies can therefore be the focus of a specific systematic review, aiming to identify which intervention (i.e. construction of the Technosol) allows similar or better plant growth compared to a reference soil.

**Table 3:**
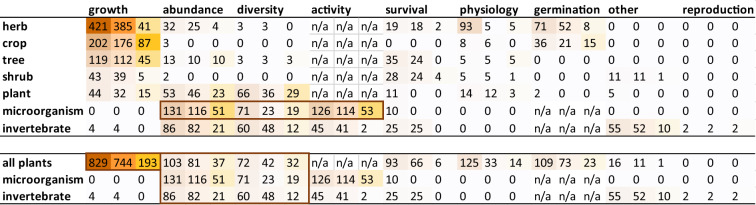
Heatmap of the distribution of studies into taxa and outcomes categories

The total number of studies is first indicated (brown shading), then the number of studies with data for quantitative and/or narrative synthesis (orange shading), and then the number of studies with data for quantitative and/or narrative synthesis and with comparison with a reference soil (yellow shading). Some outcomes do not apply to certain taxa (n/a)

Another specific systematic review on the effect of compost addition on plant growth may also be of interest to managers. To this aim, a subset of 125 studies can be selected from the cluster, having data for quantitative and/or narrative synthesis, and dealing with Technosols constructed from mineral waste and compost, which can be compared with a Technosol constructed from mineral waste only.

#### What is the potential of Technosols constructed from mineral wastes to support biodiversity?

A second knowledge cluster of 523 studies addresses the second and third most studied outcomes (abundance and diversity) for all biological groups (Table 3). It is reduced to 392 studies with data for quantitative and/or narrative synthesis, and 172 studies comparing taxa abundance or diversity between a Technosol constructed from mineral waste and a reference soil. These studies can therefore also be the focus of a specific systematic review, aiming to identify the intervention (i.e. construction of the Technosol) that results in an abundance or diversity of taxa comparable to a reference soil.

#### How do microbial communities develop in Technosols constructed from mineral wastes?

A third knowledge cluster of 328 studies addresses microorganisms, mainly measuring their abundance, diversity and activity (Table 3). It is reduced to 253 studies with data for quantitative and/or narrative synthesis, and 123 studies comparing microorganism's abundance, diversity or activity between a Technosol constructed from mineral waste and a reference soil. These studies can therefore also be the focus of a specific systematic review, aimed at identifying the intervention (i.e. construction of the Technosol) that makes it possible to obtain a microbial community comparable to that of a reference soil.

### Knowledge gaps

#### Geographical regions

The African continent appeared understudied, with only one article from South Africa (Fig. 4).

#### Taxa

The organisms studied were limited to the first organisms colonising the soil, namely microorganisms, soil invertebrates and plants (Table 3). No vertebrates were recorded. Furthermore, most of the articles assessed the response of only one biological group (plants 53.6%, microorganisms 20.3% or invertebrates 5.9%) and very few have attempted to assess the functionality of constructed soils for several biological groups at the same time (3.9%).

#### Outcome

The studies mainly assessed whether organisms could live in constructed Technosols, but very few studies (only two) examined whether organisms could also reproduce there (Table 3).

#### Time after intervention

The potential of Technosols constructed from mineral wastes for biodiversity has yet to be assessed in the long term, as only 8.3% of the studies made this assessment after 10 years or more (Table 2).

### Limitations of the map

#### Limitations of the synthesis method

Screening of articles and coding of studies were not performed independently by two reviewers, and errors in the classification of articles or studies may therefore have occurred. Nevertheless, an assessment of the agreement between the reviewers was carefully done beforehand, and we are therefore confident that the risk of excluding relevant articles or misclassifying studies is minimised.

Another limitation relates to the search string, which combines keywords describing constructed Technosols with keywords describing some of the mineral waste used to construct the Technosols. As the company that funded the systematic map specialises in the storage of inert materials from building and public works sites, articles describing Technosols constructed from these mineral wastes were particularly searched and the corresponding keywords were included in the search string. Other types of mineral waste such as mine waste, industrial waste or sediment were however included in the systematic map, but the specific corresponding keywords were not included in the search string and these categories might therefore be underrepresented. However, we are convinced that this underrepresentation is very limited, as these articles would normally include keywords related to constructed Technosols that are present in the search string.

One more limitation relates to the diversity of terms used to describe soils constructed from mineral waste. The international soil classification [9] classifies them as constructed Technosols, but the term Technosols, adopted in 2006 [22], is not always used. For example, of the 153 articles included in the systematic map, 55 articles, i.e. more than a third, never used the term “Technosols”. For this reason, we have included in the search string terms used in many classification systems such as “Anthrosols”, “Anthroposols” or “anthropogenic soils” [23], as well as general terms describing constructed soils, but studies using specific classification systems (e.g. “human transported material” in the United States Department of Agriculture’s system) or describing constructed soils from mineral waste only as urban soils or reclamation soils, may have been missed.

Finally, the main search for evidence was conducted in English, and evidence published in non-English languages other than French may have been missed. Non-English language literature can be a substantial source of evidence [24], as illustrated by the high number of articles written in French that we found. In addition, when we checked whether articles excluded because of language answered the review question, this was the case for three articles (one in Chinese, one in German and one in Portuguese), indicating that more evidence on this question is available in other languages.

#### Limitations of the evidence base

The main limitation of the existing evidence on the potential of Technosols constructed from mineral wastes to support biodiversity is that it is limited to the first organisms colonising the soil, and an overall assessment of the capacity of these Technosols to support an ecosystem is lacking. Indeed, very few articles (3.9%) have attempted to assess the functionality of constructed Technosols for several biological groups at the same time.

Likewise, the evidence on the potential of Technosols constructed from mineral wastes to support biodiversity in the long-term (> 10 years) is limited (8.3% of the studies).

## Conclusions

This systematic map showed evidence on the potential of soils constructed from mineral wastes to support biodiversity from 153 articles corresponding to 1962 studies (a study being a combination of a taxon, an intervention and an outcome). Three main knowledge clusters were identified which could be addressed in the future for full synthesis of the results (i.e. systematic reviews): (1) How well do plants grow in Technosols constructed from mineral wastes? (2) What is the potential of Technosols constructed from mineral wastes to support biodiversity (measured as abundance and diversity)? and (3) How do microbial communities develop in Technosols constructed from mineral wastes?

### Implication for policy/management

The database provided by this systematic map references the materials and their organisation for the construction of Technosols from mineral waste. This map will therefore be useful for stakeholders engaged in a circular economy approach to identify relevant studies for the construction of their soil from mineral waste. Managers will also be able to identify the research groups working on this relatively new field of research, to foster cooperation between research and practical application in the field. Thus, developing pedological engineering in urban areas can contribute to testing new solutions for the future of the interactions between human and ecological systems [25]. Given the limitations of the existing evidence described above, such cooperation would allow a more thorough and long-term assessment of the biodiversity response to Technosols constructed from mineral wastes. This map can also help to identify mineral material of interest which can improve recycling rates, reduce mineral waste production, and lower the consumption of primary resources for soil reclamation in the cities (e.g. [26]). The diversification of materials may contribute to the creation of low-cost, functional soil-like substrates able to support biodiversity as an alternative to the use of topsoil from agricultural and natural areas. Pedological engineering also offer new horizons for urban agriculture by mitigating plant contamination in a polluted context [27, 28].

### Implication for research

The systematic map also highlighted several knowledge gaps, and the following research directions for the future were identified. First, biodiversity response to Technosols construction from mineral waste should be assessed on several biological groups simultaneously (e.g. microorganisms, plants, invertebrates, and vertebrates) to better inform on the capacity of these Technosols to support a functional ecosystem. Indeed, assessing only whether plants are capable of growing in the constructed soil, as is most often done, does not tell whether a functional ecosystem will develop in the future. Plants, aboveground and belowground communities are interconnected and a group of organisms on one side of the aboveground-belowground interface can often exert positive, neutral, or negative effects on the other side of the interface, depending on context [29, 30]. These biotic interactions can also be used in soil construction by directly incorporating soil microorganisms (e.g. [31, 32]) or invertebrates (e.g. [33], [34]) to maximize internal ecological processes and positive feedbacks between the aboveground and belowground compartments over time (e.g. [35]). Second, the evaluation of biodiversity dynamics in constructed Technosols will require setting up and monitoring in situ experiments over a long period (e.g. more than 10 years). Another research question is to identify the most relevant mineral and organic wastes to construct a soil that is optimal to support biodiversity and adapted to local conditions. This point could be challenging as wastes and by-products that are generated by the urban system are extremely diverse. Thus, future research works should focus on the prediction and modelling of the emerging properties of the parent materials mixtures that will shape the habitat for biodiversity (e.g. [36]). How the different materials are combined and in particular what proportion of organic matter in addition to mineral waste maximises the potential of constructed Technosols for biodiversity should also be explored (e.g. [37]). Finally, as the potential of soils constructed from mineral wastes to support biodiversity is an emerging issue that research is only beginning to address (more than two-thirds of the literature was produced since 2015), it would be useful to update this systematic map in the near future.

## Supplementary Information

**Additional file 1. **ROSES systematic map checklist. ROSES form for systematic map reports version 1.0.

**Additional file 2. **Scoping exercise. Details of the scoping exercise to build the search string and test list used to assess the comprehensiveness.

**Additional file 3. **Search on organisational websites. Details and results of the search on organisational websites.

**Additional file 4. **List of excluded articles. List of articles excluded at title/abstract screening and excluded at full-text screening with reasons.

**Additional file 5. **Systematic map database. Coding book; list of articles included in the map; and list of the studies with their descriptors.

## Data Availability

All data generated or analysed during this study are included in this published article and its supplementary information files.

## References

[1] United Nations. The World’s Cities in 2018 - Data booklet. United Nations; 2018. 31 p. (Statistical Papers - United Nations (Ser. A), Population and Vital Statistics Report).

[2] Wilson DC, Velis CA. Waste management – still a global challenge in the 21st century: An evidence-based call for action. Waste Manag Res. 2015;33(12):1049–51. 26574579 10.1177/0734242X15616055

[3] Krook J, Svensson N, Eklund M. Landfill mining: A critical review of two decades of research. Waste Manag. 2012;32(3):513–20. 22083108 10.1016/j.wasman.2011.10.015

[4] Bolund P, Hunhammar S. Ecosystem services in urban areas. Ecol Econ. 1999;29(2):293–301. 10.1016/S0921-8009(99)00013-0

[5] Stockmann U, Minasny B, McBratney AB. How fast does soil grow? Geoderma. 2014;216:48–61. 10.1016/j.geoderma.2013.10.007

[6] Séré G, Schwartz C, Ouvrard S, Sauvage C, Renat JC, Morel JL. Soil construction: A step for ecological reclamation of derelict lands. J Soils Sediments. 2008;8(2):130–6. 10.1065/jss2008.03.277

[7] Morel JL, Chenu C, Lorenz K. Ecosystem services provided by soils of urban, industrial, traffic, mining, and military areas (SUITMAs). J Soils Sediments. 2015;15(8):1659–66. 10.1007/s11368-014-0926-0

[8] Vasenev VI, Van Oudenhoven APE, Romzaykina ON, Hajiaghaeva RA. The ecological functions and ecosystem services of urban and technogenic soils: from theory to practice (a review). Eurasian Soil Sci. 2018;51(10):1119–32. 10.1134/S1064229318100137

[9] Food and Agriculture Organization of the United Nations. World reference base for soil resources 2014: international soil classification system for naming soils and creating legends for soil maps. Rome: FAO; 2014.

[10] Prokofyeva TV, Martynenko IA, Ivannikov FA. Classification of Moscow soils and parent materials and its possible inclusion in the classification system of Russian soils. Eurasian Soil Sci. 2011;44(5):561–71. 10.1134/S1064229311050127

[11] Rodríguez-Espinosa T, Navarro-Pedreño J, Gómez-Lucas I, Jordán-Vidal MM, Bech-Borras J, Zorpas AA. Urban areas, human health and technosols for the green deal. Environ Geochem Health. 2021;43(12):5065–86. 33945056 10.1007/s10653-021-00953-8 PMC8093134

[12] Deeb M, Groffman PM, Blouin M, Egendorf SP, Vergnes A, Vasenev V, et al. Using constructed soils for green infrastructure – challenges and limitations. SOIL. 2020;6(2):413–34. 10.5194/soil-6-413-2020

[13] Fabbri D, Pizzol R, Calza P, Malandrino M, Gaggero E, Padoan E, et al. Constructed technosols: a strategy toward a circular economy. Appl Sci. 2021;11(8):3432. 10.3390/app11083432

[14] Oliveira Gonçalves J, Fruto CM, Barranco MJ, Oliveira MLS, Ramos CG. Recovery of degraded areas through technosols and mineral nanoparticles: a review. Sustainability. 2022;14(2):993. 10.3390/su14020993

[15] Pullin AS, Frampton GK, Livoreil B, Petrokofsky G, éditeurs. Guidelines and standards for evidence synthesis in environmental management. Version 5.1. 2022.

[16] Haddaway NR, Macura B, Whaley P, Pullin AS. ROSES for Systematic Map Reports. Version 1.0. 2017.

[17] Ouédraogo DY, Sordello R, Reyjol Y, Lerch T. What evidence exists on the potential of Technosols constructed from mineral wastes to host biodiversity?: a Systematic Map Protocol. PROCEED-22–00018. 10.57808/proceed.2022.3. 2022;

[18] Lopez T. Synthèse de 10 ans de recherche sur les Technosols. 2020.

[19] Kohl C, McIntosh EJ, Unger S, Haddaway NR, Kecke S, Schiemann J, et al. Online tools supporting the conduct and reporting of systematic reviews and systematic maps: a case study on CADIMA and review of existing tools. Environ Evid. 2018;7(1):8. 10.1186/s13750-018-0115-5

[20] van Eck NJ, Waltman L. Software survey: VOSviewer, a computer program for bibliometric mapping. Scientometrics. 2010;84(2):523–38. 20585380 10.1007/s11192-009-0146-3 PMC2883932

[21] Haddaway NR, Macura B, Whaley P, Pullin AS. ROSES flow diagram for systematic maps. Version 1.0. 2017

[22] Schad P. Technosols in the World Reference Base for Soil Resources–history and definitions. Soil Sci Plant Nutr. 2018;64(2):138–44. 10.1080/00380768.2018.1432973

[23] Naeth MA, Leskiw LA, Brierley JA, Warren CJ, Keys K, Dlusskiy K, et al. Revised proposed classification for human modified soils in Canada: Anthroposolic order. Can J Soil Sci. 2023;103(1):81–102. 10.1139/cjss-2022-0033

[24] Amano T, Berdejo-Espinola V, Christie AP, Willott K, Akasaka M, Báldi A, et al. Tapping into non-English-language science for the conservation of global biodiversity. PLOS Biol. 2021;19(10): e3001296. 34618803 10.1371/journal.pbio.3001296 PMC8496809

[25] Barot S, Abbadie L, Auclerc A, Barthélémy C, Bérille E, Billet P, et al. Urban ecology, stakeholders and the future of ecology. Sci Total Environ. 2019;667:475–84. 30833246 10.1016/j.scitotenv.2019.02.410

[26] Rokia S, Séré G, Schwartz C, Deeb M, Fournier F, Nehls T, et al. Modelling agronomic properties of Technosols constructed with urban wastes. Waste Manag. 2014;34(11):2155–62. 24418669 10.1016/j.wasman.2013.12.016

[27] Barbillon A, Lerch TZ, Araujo JHR, Manouchehri N, Robain H, Pando-Bahuon A, et al. Recycling wastes to mitigate trace elements contamination in plants: a new horizon for urban agriculture in polluted soils. Front Soil Sci. 2023;3:89. 10.3389/fsoil.2023.1163356

[28] Egendorf SP, Cheng Z, Deeb M, Flores V, Paltseva A, Walsh D, et al. Constructed soils for mitigating lead (Pb) exposure and promoting urban community gardening: The New York City Clean Soil Bank pilot study. Landsc Urban Plan. 2018;175:184–94. 10.1016/j.landurbplan.2018.03.012

[29] van der Putten WH, Bardgett RD, Bever JD, Bezemer TM, Casper BB, Fukami T, et al. Plant–soil feedbacks: the past, the present and future challenges. J Ecol. 2013;101(2):265–76. 10.1111/1365-2745.12054

[30] Wardle DA, Bardgett RD, Klironomos JN, Setälä H, van der Putten WH, Wall DH. Ecological linkages between aboveground and belowground biota. Science. 2004;304(5677):1629–33. 15192218 10.1126/science.1094875

[31] Cardinale M, Brusetti L, Lanza A, Orlando S, Daffonchio D, Puglia AM, et al. Rehabilitation of Mediterranean anthropogenic soils using symbiotic wild legume shrubs: Plant establishment and impact on the soil bacterial community structure. Appl Soil Ecol. 2010;46(1):1–8. 10.1016/j.apsoil.2010.05.007

[32] Valero N, Melgarejo LM, Ramírez R. Effect of low-rank coal inoculated with coal solubilizing bacteria on edaphic materials used in post-coal-mining land reclamation: A greenhouse trial. Chem Biol Technol Agric. 2016;3(1):89. 10.1186/s40538-016-0068-2

[33] Araujo JHR, Pando-Bahuon A, Hartmann C, Aroui-Boukbida H, Desjardins T, Lerch TZ. Making Green(s) With Black and White: Constructing Soils for Urban Agriculture Using Earthworms, Organic and Mineral Wastes. Front Ecol Evol. 2022;10:8. 10.3389/fevo.2022.884134

[34] Auclerc A, Beaumelle L, Barantal S, Chauvat M, Cortet J, De Almeida T, et al. Fostering the use of soil invertebrate traits to restore ecosystem functioning. Geoderma. 2022;424: 116019. 10.1016/j.geoderma.2022.116019

[35] Araujo JHR, Mikajlo I, Lerch TZ. Introduction of earthworms into constructed soils has long-lasting effects on primary production. Eur J Soil Biol. 2023;118: 103538. 10.1016/j.ejsobi.2023.103538

[36] Leguédois S, Séré G, Auclerc A, Cortet J, Huot H, Ouvrard S, et al. Modelling pedogenesis of Technosols. Geoderma. 2016;262:199–212. 10.1016/j.geoderma.2015.08.008

[37] Pruvost C, Mathieu J, Nunan N, Gigon A, Pando A, Lerch TZ, et al. Tree growth and macrofauna colonization in Technosols constructed from recycled urban wastes. Ecol Eng. 2020;153: 105886. 10.1016/j.ecoleng.2020.105886

