# Supplementary material for: Existing evidence on the potential of soils constructed from mineral wastes to support biodiversity: a systematic map
Source: Environ Evid. 2024 Apr 8;13:9. doi: 10.1186/s13750-024-00332-7 (PMC11378810; doi:10.1186/s13750-024-00332-7)
Supplement: Supplementary file 2 — Additional file 2. Scoping exercise. Details of the scoping exercise to build the search string and test list used to assess the comprehensiveness. [file 13750_2024_332_MOESM2_ESM.docx]

**Additional File 2: Scoping exercise.** Details of the scoping exercise to build the search string (Table A) and test list used to assess the comprehensiveness (Table B).

**Table A**. Details of the scoping exercise to build the search string. It was done in the Web of Science Core Collection (WOSCC) database, accessed through a CNRS (the French National Centre for Scientific Research) subscription with the following indexes: Science Citation Index Expanded (SCI-EXPANDED)--1900-present; Social Sciences Citation Index (SSCI)--1956-present; Arts & Humanities Citation Index (AHCI)--1975-present; Conference Proceedings Citation Index – Science (CPCI-S)--1998-present; Conference Proceedings Citation Index – Social Science & Humanities (CPCI-SSH)--1998-present; Emerging Sources Citation Index (ESCI)--2015-present; Current Chemical Reactions (CCR-EXPANDED)--1985-present; Index Chemicus (IC)--1993-present. The column “PICO” shows the elements of the question that are described by the keywords (P = population, I = intervention, C = comparator, O = outcome). The column “Comprehensiveness” shows the proportion of articles in the test list and indexed in WOSCC that were retrieved by the search string.

| **Step** | **PICO** | **Search string number** | **Search string** | **Number of hits** | **Comprehensiveness** | **Comments** |
| --- | --- | --- | --- | --- | --- | --- |
| Preliminary steps (10 May 2022): A test list of 15 articles was established based on the reference of published reviews, of which 14 were indexed in WOSCC. | | | | | | |
| 0 | Not applicable | Test list 1 | DO=(10.1007/s11368-021-03112-9 OR 10.1016/j.apsoil.2017.12.001 OR 10.1007/s12665-018-7848-x OR 10.1016/j.landurbplan.2018.03.012 OR 10.1016/j.jhazmat.2012.06.041 OR 10.1007/s12649-019-00822-7 OR 10.1016/j.ecoleng.2014.03.039 OR 10.1016/j.ecoleng.2020.105886 OR 10.1016/j.ecoleng.2021.106174 OR 10.1065/jss2008.03.277 OR 10.1016/j.iswcr.2019.04.002 OR 10.1016/j.jenvman.2020.111168 OR 10.1016/j.landurbplan.2016.10.002 OR 10.1007/s11368-016-1524-0) OR TI=("Potential of Biodiversity in the construction of Technosols from urban waste") | 14 | Not applicable | PhD thesis not indexed in WOSCC |
| 1 | I | #1 | TS=(technosol$ OR technosoil$ OR techno-soil$ OR "construct* soil$" OR "engineered soil$" OR "rebuilt soil$") | 910 | 14/14 |  |
| 2 | I | #1a | TS=(technosol$ OR technosoil$ OR techno-soil$ OR "construct* soil$" OR "engineered soil$" OR "rebuilt soil$" OR "artificial soil$") | 2 165 | 14/14 | Adding "artificial soil$": Rapid screening on title of the first 100 articles of the 1255 articles added (sorted by relevance) indicates that these articles may be in scope |
| 3 | I | #1b | TS=(technosol$ OR technosoil$ OR techno-soil$ OR "construct* soil$" OR "engineered soil$" OR "rebuilt soil$" OR "artificial soil$" OR "urban soil$" OR anthroposol$ OR "anthropogenic soil$") | 6 115 | 14/14 | Adding "urban soil$" OR anthroposol$ OR "anthropogenic soil$" |
| 4 | I | #1c | TS=(technosol$ OR technosoil$ OR techno-soil$ OR "construct* soil$" OR "engineered soil$" OR "rebuilt soil$" OR "artificial soil$" OR "urban soil$" OR anthroposol$ OR "anthropogenic soil$" OR "structural soil$") | 6 246 | 14/14 | Adding "structural soil$" |
| 5 | I | #2 | TS=((*soil$ OR earth OR horizon$ OR material$ OR sediment$) AND (concrete OR clinker OR brick$ OR ballast OR waste$ OR debris OR excavat* OR demolition OR rubble OR inert OR industrial OR *contaminat* OR manufactur* OR mining)) | 697 483 | 12/14 | Enable to capture 590 out of the 910 articles retrieved with #1. Looking at the 320 articles, keywords « artificial soil, urban soil, anthroposol, anthropogenic soil, structural soil » were identified |
| 6 | I | #2a | TS=((*soil$ OR earth OR horizon$ OR material$ OR sediment$) AND (concrete OR clinker OR brick$ OR ballast OR backfill$ OR waste$ OR debris OR excavat* OR demolition OR rubble OR inert OR industrial OR *contaminat* OR manufactur* OR mining OR urban)) | 739 620 | 14/14 | Adding backfill$ and urban |
| 7 | I | #2b | TS=((*soil$ OR earth OR horizon$ OR sediment$) AND (concrete OR clinker OR brick$ OR ballast OR backfill$ OR waste$ OR debris OR excavat* OR demolition OR rubble OR inert OR industrial OR *contaminat* OR manufactur* OR mining OR urban)) | 284 482 | 14/14 | Removing material$ increases specificity.  Rapid screening on title of the first 100 articles of the 455 138 articles removed (sorted by relevance) indicates that these articles were about using wastes to make construction material, road, etc. (circular economy in the construction sector) |
| 8 | I | #2c | TS=((*soil$ OR earth OR horizon$) AND (concrete OR clinker OR brick$ OR ballast OR backfill$ OR waste$ OR debris OR excavat* OR demolition OR rubble OR inert OR industrial OR *contaminat* OR manufactur* OR mining OR urban OR sediment$)) | 284 413 | 14/14 | Moving sediment$ |
| 9 | I | #2d | TS=((*soil$ OR earth OR horizon$) AND (concrete OR clinker OR brick$ OR ballast OR backfill$ OR waste$ OR debris OR excavat* OR demolition OR rubble OR inert OR industrial OR *contaminat* OR manufactur* OR mining OR urban)) | 229 442 | 14/14 | Removing sediment$  #2c NOT #2d Rapid screening on title of the first 100 articles of the 54 971 articles removed (sorted by relevance) indicates that these articles were about soil characteritics/composition  #2b NOT #2d Rapid screening on title of the first 100 articles of the 55 040 articles removed (sorted by relevance) indicates that these articles were about using sediment in cement, brick production, etc. as construction material, also contamination of sediment |
| 10 | I | #2e | TS=((*soil$ OR earth) AND (concrete OR clinker OR brick$ OR ballast OR backfill$ OR waste$ OR debris OR excavat* OR demolition OR rubble OR inert OR industrial OR *contaminat* OR manufactur* OR mining OR urban)) | 220 051 | 14/14 | Removing horizon$  #2d NOT #2e Rapid screening on title of the first 100 articles of the 9 391 articles removed (sorted by relevance) indicates that these articles are out of scope |
| 11 | I | #2f | TS=((*soil$) AND (concrete OR clinker OR brick$ OR ballast OR backfill$ OR waste$ OR debris OR excavat* OR demolition OR rubble OR inert OR industrial OR *contaminat* OR manufactur* OR mining OR urban)) | 187 410 | 14/14 | Removing earth  #2e NOT #2f Rapid screening on title of the first 100 articles of the 32 641 articles removed (sorted by relevance) indicates that these articles are out of scope, earth as a building material (earth-based mixtures) |
| 12 | I | #3 | TS=((*soil$) AND (concrete OR clinker OR brick$ OR ballast OR backfill$ OR waste$ OR debris OR excavat* OR rubble OR inert)) | 74 128 | 12/14 | Removing demolition OR industrial OR *contaminat* OR manufactur* OR mining OR urban)) that are too general and were used for « industrial soil », « mining waste », « demolition rubble », « manufacturing waste » 🡪 these keywords are maily captured with those present (e.g waste, rubble)  2 articles from the test list (Ugloni 2020 and Burrow 2018) are missing  -> keyword urban important |
| 13 | I | #3a | TS=((*soil$) AND (concrete OR clinker OR brick$ OR ballast OR backfill$ OR waste$ OR debris OR excavat* OR rubble OR inert OR urban)) | 95 000 | 14/14 | #3 with keyword urban |
| 14 | I | #4 | #3a OR #1c | 96 998 | 14/14 |  |
| 15 | I | #5 | TS=((technosol$ OR technosoil$ OR techno-soil$ OR "construct* soil$" OR "engineered soil$" OR "rebuilt soil$" OR "artificial soil$" OR "urban soil$" OR anthroposol$ OR "anthropogenic soil$" OR "structural soil$") OR ((*soil$) AND (concrete OR clinker OR brick$ OR ballast OR backfill$ OR waste$ OR debris OR excavat* OR rubble OR inert))) | 79 317 | 14/14 | Compared to #4, this removed *soil$ AND urban  #4 NOT #5 (effect of removing *soil$ AND urban): a rapid screening of the 17 681 articles indicates that they are out of scope (urbanisation effect on soil) |
| 16 | I | #5a | TS=((technosol$ OR technosoil$ OR techno-soil$ OR "construct* soil$" OR "engineered soil$" OR "rebuilt soil$" OR "artificial soil$" OR "urban soil$" OR anthroposol$ OR "anthropogenic soil$" OR "structural soil$") OR ((soil$ OR topsoil$ OR subsoil$) AND (concrete OR clinker OR brick$ OR ballast OR backfill$ OR waste$ OR debris OR excavat* OR rubble OR inert))) | 79 353 | 14/14 | Adding topsoil$ OR subsoil$: a rapid screening of the 137 articles added indicates that they are out of scope |
| 17 | P | #6 | TS=(biodiversity OR species OR organism$ habitat$ OR ecosystem$ OR flora OR plant$ OR vegetation OR tree$ OR crop$ OR *grass* OR weed$ OR forest$ OR wood* OR *fauna OR animal$ OR invertebrate$ OR vertebrate$ OR insect$ OR arthropod$ OR arachnid$ OR collembola OR coleoptera OR beetle$ OR nematod$ OR fung* OR microb* OR bacter*  OR ant$ OR earthworm$) | 8 735 429 | 14/14 |  |
| 18 | P & I | #7 | #5 AND #6 | 40 008 | 14/14 |  |
| 19 | P & I | #8 | #1c AND #6 | 3 617 | 14/14 |  |
| 19 | P & I | #8 | TS=((technosol$ OR technosoil$ OR techno-soil$ OR "construct* soil$" OR "engineered soil$" OR "rebuilt soil$" OR "artificial soil$" OR "urban soil$" OR anthroposol$ OR "anthropogenic soil$" OR "structural soil$") AND (biodiversity OR species OR organism$ habitat$ OR ecosystem$ OR flora OR plant$ OR vegetation OR tree$ OR crop$ OR *grass* OR weed$ OR forest$ OR wood* OR *fauna OR animal$ OR invertebrate$ OR vertebrate$ OR insect$ OR arthropod$ OR arachnid$ OR collembola OR coleoptera OR beetle$ OR nematod$ OR fung* OR microb* OR bacter* OR ant$ OR earthworm$)) | 3 617 | 14/14 |  |
| 20 | I | #1d | TS=(technosol$ OR technosoil$ OR techno-soil$ OR "construct* soil$" OR "engineered soil$" OR "rebuilt soil$" OR "artificial soil$" OR "urban soil$" OR anthroposol$ OR "anthropogenic soil$" OR "structural soil$" OR concrete OR clinker OR brick$ OR ballast OR backfill$ OR "demolition waste$" OR "manufacturing waste$" OR "mining waste$" OR "urban waste$" OR debris OR excavat* OR rubble OR inert) | 548 277 | 14/14 |  |
| 21 | P & I | #9 | #1d AND #6 | 81 441 | 14/14 |  |
| 22 | P & I | #9a | TS=((technosol$ OR technosoil$ OR techno-soil$ OR "construct* soil$" OR "engineered soil$" OR "rebuilt soil$" OR "artificial soil$" OR "urban soil$" OR anthroposol$ OR "anthropogenic soil$" OR "structural soil$" OR "backfill$") AND (biodiversity OR species OR organism$ habitat$ OR ecosystem$ OR flora OR plant$ OR vegetation OR tree$ OR crop$ OR *grass* OR weed$ OR forest$ OR wood* OR *fauna OR animal$ OR invertebrate$ OR vertebrate$ OR insect$ OR arthropod$ OR arachnid$ OR collembola OR coleoptera OR beetle$ OR nematod$ OR fung* OR microb* OR bacter* OR ant$ OR earthworm$)) | 4 169 | 14/14 | Adding backfill$  Rapid screening on title of the first 100 articles of the 552 articles removed (sorted by relevance) indicates that these articles were about horticulture-plantation technics (backfill composition, amendments when planting) or mining backfill – backfill paste plants |
| 23 | P & I | #9b | TS=((technosol$ OR technosoil$ OR techno-soil$ OR "construct* soil$" OR "engineered soil$" OR "rebuilt soil$" OR "artificial soil$" OR "urban soil$" OR anthroposol$ OR "anthropogenic soil$" OR "structural soil$" OR "excavated soil$" OR "inert soil$") AND (biodiversity OR species OR organism$ habitat$ OR ecosystem$ OR flora OR plant$ OR vegetation OR tree$ OR crop$ OR *grass* OR weed$ OR forest$ OR wood* OR *fauna OR animal$ OR invertebrate$ OR vertebrate$ OR insect$ OR arthropod$ OR arachnid$ OR collembola OR coleoptera OR beetle$ OR nematod$ OR fung* OR microb* OR bacter* OR ant$ OR earthworm$)) | 3749 | 14/14 | Adding "excavated soil$" OR "inert soil$"  Rapid screening on title of the first 100 articles of the 132 articles removed (sorted by relevance) indicates that these articles were about assessing the suitability of excavated soil as a by-product (for further use), ecotoxicity studies |
| 24 | P & I | #9c | TS=((technosol$ OR technosoil$ OR techno-soil$ OR "construct* soil$" OR "engineered soil$" OR "rebuilt soil$" OR "artificial soil$" OR "urban soil$" OR anthroposol$ OR "anthropogenic soil$" OR "structur* soil$") AND (biodiversity OR species OR organism$ habitat$ OR ecosystem$ OR flora OR plant$ OR vegetation OR tree$ OR crop$ OR *grass* OR weed$ OR forest$ OR wood* OR *fauna OR animal$ OR invertebrate$ OR vertebrate$ OR insect$ OR arthropod$ OR arachnid$ OR collembola OR coleoptera OR nematod$ OR fung* OR microb* OR bacter*  OR ant$ OR earthworm$ OR beetle$)) | 4 273 | 14/14 | Change structural soil$ in structur* soil to include structured soil -> add 656 articles  🡪 NO « structured soil » not in scope |
| 25 | P & I | #9d | TS=((technosol$ OR technosoil$ OR techno-soil$ OR "construct* soil$" OR "engineered soil$" OR "rebuilt soil$" OR "artificial soil$" OR "urban soil$" OR anthroposol$ OR "anthropogenic soil$" OR "structural soil$" OR "excavated soil$" OR "inert soil$" OR "fabricated soil$" OR "excavated material$" OR "excavated earth" OR "inert material$") AND (biodiversity OR species OR organism$ habitat$ OR ecosystem$ OR flora OR plant$ OR vegetation OR tree$ OR crop$ OR *grass* OR weed$ OR forest$ OR wood* OR *fauna OR animal$ OR invertebrate$ OR vertebrate$ OR insect$ OR arthropod$ OR arachnid$ OR collembola OR coleoptera OR nematod$ OR fung* OR microb* OR bacter* OR ant$ OR earthworm$ OR beetle$)) | 4 233 | 14/14 | Adding "fabricated soil$" OR "excavated material$" OR "excavated earth" OR "inert material$"  Add 484 articles |
| 26 | P & I | #10a | TS=((technosol$ OR technosoil$ OR techno-soil$ OR "construct* soil$" OR "engineered soil$" OR "rebuilt soil$" OR "artificial soil$" OR "urban soil$" OR anthroposol$ OR "anthropogenic soil$" OR "structural soil$" OR "fabricated soil$") AND (biodiversity OR species OR organism$ OR habitat$ OR ecosystem$ OR flora OR plant$ OR vegetation OR tree$ OR crop$ OR *grass* OR weed$ OR forest$ OR wood* OR *fauna OR animal$ OR invertebrate$ OR vertebrate$ OR insect$ OR arthropod$ OR arachnid$ OR collembola OR coleoptera OR nematod$ OR fung* OR microb* OR bacter* OR ant$ OR earthworm$ OR beetle$)) | 3 620 | 14/14 | Preliminary final search string 1 (reduced) Correction error OR between organism$ habitat |
| 27 | P & I | #10b | TS=((technosol$ OR technosoil$ OR techno-soil$ OR "construct* soil$" OR "engineered soil$" OR "rebuilt soil$" OR "artificial soil$" OR "urban soil$" OR anthroposol$ OR "anthropogenic soil$" OR "structural soil$" OR "fabricated soil$" OR "excavated soil$" OR "inert soil$" OR "excavated material$" OR "excavated earth" OR "inert material$" OR backfill$) AND (biodiversity OR species OR organism$ OR habitat$ OR ecosystem$ OR flora OR plant$ OR vegetation OR tree$ OR crop$ OR *grass* OR weed$ OR forest$ OR wood* OR *fauna OR animal$ OR invertebrate$ OR vertebrate$ OR insect$ OR arthropod$ OR arachnid$ OR collembola OR coleoptera OR nematod$ OR fung* OR microb* OR bacter* OR ant$ OR earthworm$ OR beetle$)) | 4 829 | 14/14 | Preliminary final search string 2 (full). Correction error OR between organism$ habitat |
| The two search strings #10a and #10b were discussed with members of the ECT company, and it was decided to focus on only one block of keywords for intervention (constructed Technosols). The following set of tests for the search string was then conducted (19 May 2022), starting from the block intervention of the search string #10b. | | | | | | |
| 28 | I | #11 | TS=(technosol$ OR technosoil$ OR techno-soil$ OR "construct* soil$" OR "engineered soil$" OR "rebuilt soil$" OR "artificial soil$" OR "urban soil$" OR anthroposol$ OR "anthropogenic soil$" OR "structural soil$" OR "fabricated soil$" OR "excavated soil$" OR "inert soil$" OR "excavated material$" OR "excavated earth" OR "inert material$" OR backfill$) | 15 534 | 14/14 |  |
| 29 | I | #11a | TS=(technosol$ OR technosoil$ OR techno-soil$ OR "construct* soil$" OR "engineered soil$" OR "rebuilt soil$" OR "artificial soil$" OR "urban soil$" OR anthroposol$ OR "anthropogenic soil$" OR "structural soil$" OR "fabricated soil$" OR "excavated soil$" OR "inert soil$" OR "excavated material$" OR "excavated earth" OR "inert material$") | 8 541 | 14/14 | Removing backfill$ reduced the number of hits by almost two |
| 30 | I | #12 | TS=(backfill$) | 7024 | 1/14 | The term brings a lot of noise (cemented backfill, mine backfill, paste backfill, backfill body, etc.) |
| 31 | I | #12a | TS=(backfill$ AND (urban)) | 81 | 1/14 | Combination of backfill and usage |
| 32 | I | #12b | TS=(backfill$ AND (urban OR green OR park$)) | 198 | 1/14 |  |
| 33 | I | #12c | TS=(backfill$ AND (urban OR green OR park$ OR lawn$ OR farm* OR agriculture OR  biodiversity OR species OR organism$ OR habitat$ OR ecosystem$ OR flora OR plant$ OR vegetation OR tree$ OR crop$ OR *grass* OR weed$ OR forest$ OR wood* OR *fauna OR animal$ OR invertebrate$ OR vertebrate$ OR fung* OR microb* OR bacter*)) | 708 | 1/14 | Screening of the first 100 hits sorted by relevance revealed that none of the titles were in scope. |
| 34 | I | #12d | TS=(backfill$ AND (technosol$)) | 2 | 1/14 |  |
| 35 | I | #11b | TS=(technosol$ OR technosoil$ OR techno-soil$ OR "construct* soil$" OR "engineered soil$" OR "rebuilt soil$" OR "artificial soil$" OR anthroposol$ OR "anthropogenic soil$" OR "structural soil$" OR "fabricated soil$" OR "excavated soil$" OR "inert soil$" OR "excavated material$" OR "excavated earth" OR "inert material$") | 5 090 | 14/14 | Rapid screening of some titles of #11a showed that many irrelevant articles contain the keyword « urban » so #11b = #11a without urban soil$ that are out of scope.  Screening of the first 100 titles removed (of 3 451 sorted by relevance) => all are irrelevant |
| The search string #11b and the relevance of the search terms "backfill$" and "urban soil$" were then discussed with T. Lerch. Additional keywords were identified and the following set of tests for the search string was then conducted (8 June 2022), starting from the search string #11b. | | | | | | |
| 36 | I | #13 | TS=(technosol$ OR technosoil$ OR techno-soil$ OR "construct* soil$" OR "engineered soil$" OR "rebuilt soil$" OR "artificial soil$" OR anthroposol$ OR "anthropogenic soil$" OR "structural soil$" OR "fabricated soil$" OR "excavated soil$" OR "inert soil$" OR "excavated material$" OR "excavated earth" OR "inert material$" OR "surplus soil$") | 5 171 | 14/14 | Adding « surplus soil$ » 🡪 46 articles added, screened on title, some may be in scope (fate of excavacated surplus soil) |
| 37 | I | #14 | TS=(technosol$ OR technosoil$ OR techno-soil$ OR "construct* soil$" OR "engineered soil$" OR "rebuilt soil$" OR "artificial soil$" OR anthroposol$ OR "anthropogenic soil$" OR "structural soil$" OR "fabricated soil$" OR "excavated soil$" OR "inert soil$" OR "excavated material$" OR "excavated earth" OR "inert material$" OR "surplus soil$" OR "deep horizon$ of soil") | 5 171 | 14/14 | Adding « deep horizon$ of soil» 🡪 no articles added |
| 38 | I | #15 | TS=(technosol$ OR technosoil$ OR techno-soil$ OR "construct* soil$" OR "engineered soil$" OR "rebuilt soil$" OR "artificial soil$" OR anthroposol$ OR "anthropogenic soil$" OR "structural soil$" OR "fabricated soil$" OR "excavated soil$" OR "inert soil$" OR "excavated material$" OR "excavated earth" OR "inert material$" OR "surplus soil$" OR (soil$ AND "deep horizon$")) | 5276 | 14/14 | Adding (soil$ AND "deep horizon$") 🡪 this adds 105 articles, they were sorted by relevance and screened on titles 🡪 out of scope (nothing about constructed soils) |
| 39 | I | #16 | TS=(technosol$ OR technosoil$ OR techno-soil$ OR "construct* soil$" OR "engineered soil$" OR "rebuilt soil$" OR "artificial soil$" OR anthroposol$ OR "anthropogenic soil$" OR "structural soil$" OR "fabricated soil$" OR "excavated soil$" OR "inert soil$" OR "excavated material$" OR "excavated earth" OR "inert material$" OR "surplus soil$" OR "urban construction waste$") | 5189 | 14/14 | Adding "urban construction waste$" 🡪 this adds 18 articles, screened on title, some may be in scope (use of urban construction wastes) |
| After further discussion with T. Lerch, 5 additional references were added to the test list. They were identified from a MSc thesis (T. Lopez 2020) presenting a general overview of the literature on Technosols. Additional keywords were also identified and the following final set of tests for the search string was then conducted (17 June 2022), starting from the search string #16. | | | | | | |
| 40 | Not applicable | Test list 2 | DO=(10.1007/s11368-021-03112-9 OR 10.1016/j.apsoil.2017.12.001 OR 10.1007/s12665-018-7848-x OR 10.1016/j.landurbplan.2018.03.012 OR 10.1016/j.jhazmat.2012.06.041 OR 10.1007/s12649-019-00822-7 OR 10.1016/j.ecoleng.2014.03.039 OR 10.1016/j.ecoleng.2020.105886 OR 10.1016/j.ecoleng.2021.106174 OR 10.1065/jss2008.03.277 OR 10.1016/j.iswcr.2019.04.002 OR 10.1016/j.jenvman.2020.111168 OR 10.1016/j.landurbplan.2016.10.002 OR 10.1007/s11368-016-1524-0 OR 10.1007/s11368-018-2142-9 OR 10.1007/s11368-014-1008-z OR 10.12871/00021857201916 OR 10.1134/S1064229318100149 OR 10.3389/fevo.2022.884134) OR TI=("Potential of Biodiversity in the construction of Technosols from urban waste") | 19 | Not applicable | PhD thesis not indexed in WOSCC |
| 41 | I | #16 | TS=(technosol$ OR technosoil$ OR techno-soil$ OR "construct* soil$" OR "engineered soil$" OR "rebuilt soil$" OR "artificial soil$" OR anthroposol$ OR "anthropogenic soil$" OR "structural soil$" OR "fabricated soil$" OR "excavated soil$" OR "inert soil$" OR "excavated material$" OR "excavated earth" OR "inert material$" OR "surplus soil$" OR "urban construction waste$") | 5 203 | 19/19 | = #16 from previous test. All articles from the test list retrieved. |
| 42 | I | #17 | TS=(technosol$ OR technosoil$ OR techno-soil$ OR "construct* soil$" OR "engineered soil$" OR "rebuilt soil$" OR "artificial soil$" OR anthroposol$ OR "anthropogenic soil$" OR "structural soil$" OR "fabricated soil$" OR "excavated soil$" OR "inert soil$" OR "excavated material$" OR "excavated earth" OR "inert material$" OR "surplus soil$" OR "urban construction waste$" OR anthrosol$ OR anthrosoil$) | 5 709 | 19/19 | Adding Anthrosol$ OR anthrosoil$ (american terminology) |
| **43** | **I** | **Final** | **TS=(technosol$ OR technosoil$ OR techno-soil$ OR "construct* soil$" OR "engineered soil$" OR "rebuilt soil$" OR "artificial soil$" OR anthroposol$ OR anthroposoil$ OR "anthropogenic soil$" OR "structural soil$" OR "fabricated soil$" OR "excavated soil$" OR "inert soil$" OR "excavated material$" OR "excavated earth" OR "inert material$" OR "surplus soil$" OR "urban construction waste$" OR anthrosol$ OR anthrosoil$)** | 5 712 | 19/19 | Adding anthroposoil$ (as for technosol and anthrosol) |

**Table B**. Test list used to assess the comprehensiveness during the scoping exercise in Web of Science Core Collection (WOSCC) database.

| **Reference** | **Year** | **DOI** | **indexed in WOSCC (10 May 2022)** | **indexed in Scopus (10 May 2022)** | **indexed in WOSCC (17 June 2022)** | **indexed in Scopus (17 June 2022)** | **Source** |
| --- | --- | --- | --- | --- | --- | --- | --- |
| Abbruzzini, Thalita & Palomino, Lucy & Prado, B.. (2021). SOILS, SEC 1 SOIL ORGANIC MATTER DYNAMICS AND NUTRIENT CYCLING Evaluation of Technosols constructed with construction and excavation debris for greenhouse production of ornamental plants. Journal of Soils and Sediments. 10.1007/s11368-021-03112-9 | 2021 | 10.1007/s11368-021-03112-9 | Yes | Yes | Yes | Yes | Cite Pruvost et al. 2020 |
| Araujo,J. H. R., A. Pando-Bahuon, C. Hartmann, H. Aroui-Boukbida, T. Desjardins and Thomas Z. Lerch. Making Green(s) With Black and White: Constructing Soils for Urban Agriculture Using Earthworms, Organic and Mineral Wastes | 2022 | 10.3389/fevo.2022.884134 | Not applicable | Not applicable | Yes | Yes | Provided by T. Lerch |
| Burrow, C. Influence of connectivity & topsoil management practices of a constructed technosol on pedofauna colonization: A field study. Appl. Soil Ecol. 2018, 123, 416–419. | 2018 | 10.1016/j.apsoil.2017.12.001 | Yes | Yes | Yes | Yes | Review Fabbri & al. 2021 |
| Cannavo, P., Guénon, R., Galopin, G., and Vidal-Beaudet, L.: Technosols made with various urban wastes showed contrasted performance for tree development during a 3-year experiment, Environ. Earth Sci., 77, 1–13, https://doi.org/10.1007/s12665-018-7848-x, 2018. | 2018 | 10.1007/s12665-018-7848-x | Yes | Yes | Yes | Yes | Review Deeb et al. 2020 |
| Egendorf, S. P., Cheng, Z., Deeb, M., Flores, V., Paltseva, A., Walsh, D., Groffman, P., and Mielke, H. W.: Constructed soils for mitigating lead (Pb) exposure and promoting urban community gardening: The New York City Clean Soil Bank pilot study, Landsc. Urban Plan., 175, 184–194, https://doi.org/10.1016/j.landurbplan.2018.03.012, 2018. | 2018 | 10.1016/j.landurbplan.2018.03.012 | Yes | Yes | Yes | Yes | Review Deeb et al. 2020 |
| Hafeez, F., Spor, A., Breuil, M.-C., Schwartz, C., Martin-Laurent, F., and Philippot, L.: Distribution of bacteria and nitrogen-cycling microbial communities along constructed Technosol depth-profiles, J. Hazard. Mater., 231/232, 88–97, https://doi.org/10.1016/j.jhazmat.2012.06.041, 2012a. | 2012 | 10.1016/j.jhazmat.2012.06.041 | Yes | Yes | Yes | Yes | Review Deeb et al. 2020 |
| Hedde, Mickael, Johanne Nahmani, Geoffroy Sere, Apolline Auclerc, et Jerome Cortet. « Early colonization of constructed Technosols by macro-invertebrates ». Journal of Soils and Sediments 19, no 8 (août 2019): 3193‑3203. https://doi.org/10.1007/s11368-018-2142-9 | 2019 | 10.1007/s11368-018-2142-9 | Not applicable | Not applicable | Yes | Yes | Reference T. Lopez MSc thesis 2020 |
| Jangorzo, Nouhou Salifou, Francoise Watteau, Dorian Hajos, et Christophe Schwartz. « Nondestructive monitoring of the effect of biological activity on the pedogenesis of a Technosol ». Journal of Soils and Sediments 15, no 8 (août 2015): 1705‑15. https://doi.org/10.1007/s11368-014-1008-z | 2015 | 10.1007/s11368-014-1008-z | Not applicable | Not applicable | Yes | Yes | Reference T. Lopez MSc thesis 2020 |
| Manfredi, P., C. Cassinari, M. Gatti, et M. Trevisan. « Growth and yield response of tomato (Solanum lycopersicum L.) to soil reconstitution technology ». Agrochimica 63, no 1 (mars 2019): 73‑83. https://doi.org/10.12871/00021857201916 | 2019 | 10.12871/00021857201916 | Not applicable | Not applicable | Yes | Yes | Reference T. Lopez MSc thesis 2020 |
| Peruzzi, E.; Macci, C.; Doni, S.; Zelari, L.; Masciandaro, G. Co-composting as a Management Strategy for Posidonia oceanica Residues and Dredged Sediments | 2019 | 10.1007/s12649-019-00822-7 | Yes | Yes | Yes | Yes | Review Fabbri & al. 2021 |
| Pey, B., Cortet, J., Capowiez, Y., Nahmani, J., Watteau, F., and Schwartz, C.: Technosol composition affects Lumbricus terrestris surface cast composition and production, Ecol. Eng., 67, 238–247, https://doi.org/10.1016/j.ecoleng.2014.03.039, 2014. | 2014 | 10.1016/j.ecoleng.2014.03.039 | Yes | Yes | Yes | Yes | Review Deeb et al. 2020 |
| Pruvost, C.: Potentiel de la Biodiversité dans la construction de Technosols à partir de déchets urbains, Potential of Biodiversity in the construction of Technosols from urban waste, Ph.D. thesis, Université Paris-Est; École doctorale Sciences, Ingénierie et Environnement Laboratoire: IEES Paris – Institut d’Ecologie et des Sciences de l’Environnement de Paris (laboratoire), 173 pp., http://www.theses.fr/2018PESC1161, 2018. | 2018 | No DOI | No | No | No | No | Review Deeb et al. 2020 |
| Pruvost, C.; Mathieu, J.; Nunan, N.; Gigon, A.; Pando, A.; Lerch, T.Z.; Blouin, M. Tree growth and macrofauna colonization in Technosols constructed from recycled urban wastes. Ecol. Eng. 2020, 153, 105886. | 2020 | 10.1016/j.ecoleng.2020.105886 | Yes | Yes | Yes | Yes | Review Fabbri & al. 2021 |
| Santorufo, L.; Joimel, S.; Auclerc, A.; Deremiens, J.; Grisard, G.; Hedde, M.; Nahmani, J.; Pernin, C.; Cortet, J. Early colonization of constructed technosol by microarthropods. Ecol. Eng. 2021, 162, 106174. | 2021 | 10.1016/j.ecoleng.2021.106174 | Yes | Yes | Yes | Yes | Review Oliveira Gonzalves et al. 2022 |
| Séré, G., Schwartz, C., Ouvrard, S., Sauvage, C., Renat, J.-C., and Morel, J. L.: Soil construction: A step for ecological reclamation of derelict lands, J. Soils Sediments, 8, 130–136, https://doi.org/10.1065/jss2008.03.277, 2008 | 2008 | 10.1065/jss2008.03.277 | Yes | Yes | Yes | Yes | Review Deeb et al. 2020 |
| Slukovskaya, M. V., Vasenev, V. I., Ivashchenko, K. V., Morev, D. V., Drogobuzhskaya, S. V., Ivanova, L. A., and Kremenetskaya, I. P.: Technosols on mining wastes in the subarctic: Efficiency of remediation under Cu-Ni atmospheric pollution, Int. Soil Water Conserv. Res., 7, 297–307, https://doi.org/10.1016/j.iswcr.2019.04.002, 2019 | 2019 | 10.1016/j.iswcr.2019.04.002 | Yes | Yes | Yes | Yes | Review Deeb et al. 2020 |
| Ugolini, F.; Baronti, S.; Lanini, G.M.; Maienza, A.; Ungaro, F.; Calzolari, C. Assessing the influence of topsoil and technosol characteristics on plant growth for the green regeneration of urban built sites. J. Environ. Manag. 2020, 273, 111168 | 2020 | 10.1016/j.jenvman.2020.111168 | Yes | Yes | Yes | Yes | Review Fabbri & al. 2021 |
| Vergnes, A., Blouin, M., Muratet, A., Lerch, T. Z., Mendez-Millan, M., Rouelle-Castrec, M., and Dubs, F.: Initial conditions during Technosol implementation shape earthworms and ants diversity, Landsc. Urban Plan., 159, 32–41, https://doi.org/10.1016/j.landurbplan.2016.10.002, 2017. | 2017 | 10.1016/j.landurbplan.2016.10.002 | Yes | Yes | Yes | Yes | Review Deeb et al. 2020 |
| Villenave, C., G. Sere, C. Schwartz, F. Watteau, A. Jimenez, et J. Cortet. « Rapid Changes in Soil Nematodes in the First Years after Technosol Construction for the Remediation of an Industrial Wasteland ». Eurasian Soil Science 51, no 10 (octobre 2018): 1266‑73. https://doi.org/10.1134/S1064229318100149 | 2018 | 10.1134/S1064229318100149 | Not applicable | Not applicable | Yes | Yes | Reference T. Lopez MSc thesis 2020 |
| Yilmaz, D., Cannavo, P., Séré, G., Vidal-Beaudet, L., Legret, M., Damas, O., and Peyneau, P.-E.: Physical properties of structural soils containing waste materials to achieve urban greening, J. Soils Sediments, 18, 442–455, https://doi.org/10.1007/s11368-016-1524-0, 2016. | 2016 | 10.1007/s11368-016-1524-0 | Yes | Yes | Yes | Yes | Review Deeb et al. 2020 |

Deeb M, Groffman PM, Blouin M, Egendorf SP, Vergnes A, Vasenev V, et al. Using constructed soils for green infrastructure – challenges and limitations. SOIL. 2020;6(2):413‑34.

Fabbri D, Pizzol R, Calza P, Malandrino M, Gaggero E, Padoan E, et al. Constructed Technosols: A Strategy toward a Circular Economy. Applied Sciences. 2021;11(8):3432.

Lopez T. Synthèse de 10 ans de recherche sur les Technosols. 2020.

Oliveira Gonçalves J, Fruto CM, Barranco MJ, Oliveira MLS, Ramos CG. Recovery of Degraded Areas through Technosols and Mineral Nanoparticles: A Review. Sustainability. 2022;14(2):993.

Pruvost C, Mathieu J, Nunan N, Gigon A, Pando A, Lerch TZ, et al. Tree growth and macrofauna colonization in Technosols constructed from recycled urban wastes. Ecological Engineering. 2020;153:105886.
