# Supplementary material for: Existing evidence on the potential of soils constructed from mineral wastes to support biodiversity: a systematic map
Source: Environ Evid. 2024 Apr 8;13:9. doi: 10.1186/s13750-024-00332-7 (PMC11378810; doi:10.1186/s13750-024-00332-7)
Supplement: Supplementary file 3 — Additional file 3. Search on organisational websites. Details and results of the search on organisational websites. [file 13750_2024_332_MOESM3_ESM.docx]

**Additional file 3: Search on organisational websites.** Details and results of the search on organisational websites.

| **Organisational website** | **Date of search** | **Comment on search** | **Number of references found** |
| --- | --- | --- | --- |
| The Food and Agriculture Organization (FAO) (<https://www.fao.org/about/en/>) | 3/10/22 | Tab “Publications”, Toolbar “Search publications”  **Search string:**  **technosol OR technosols OR technosoil OR technosoils OR anthroposol OR anthroposols OR anthroposoil OR anthroposoils OR anthrosol OR anthrosols OR anthrosoil OR anthrosoils**  About 70 results screened on title | 2 |
| The European Circular Economy Stakeholder Platform (<https://circulareconomy.europa.eu/platform/en> ) | 3/10/22 | Tab “Knowledge Hub”, select “Knowledge” [*In this section you will find knowledge such as studies, reports, presentations and position papers… all submitted by stakeholders*]  **Keyword:**   - **Technosol: 0 result** - **Soil: 3 results**   Screening on title | 0 |
| The French Agency for Ecological Transition (ADEME) (<https://www.ademe.fr/> ) | 3/10/22 | “La librairie” [*Nos publications et études, magazines, guides pratiques et avis sont consultables et téléchargeables en ligne.* <https://librairie.ademe.fr/> ]  **Keyword:**   - **Technosol: 1 result** - **Sol: 483 results**   Screening on title | 1 |
| The French Biodiversity Agency (OFB) (<https://www.ofb.gouv.fr> ) | 4/10/22 | “Documentation” [La documentation scientifique et technique <https://professionnels.ofb.fr/fr/documentation>  Portail technique <https://professionnels.ofb.fr/tous-les-documents> ]  **Keyword:**   - **Technosol: 0 result** - **Soil: 1 result** - **Sol: 8 pages with about 6 results**   Screening on title  [Ne sont pas considérés comme "ouvrages" par exemple les nombreux rapports d'études. Pour consulter l'intégralité de toutes les productions S&T >  “portail documentaire partenarial" <https://www.documentation.eauetbiodiversite.fr/> ]  **Search string:**  **technosol OR technosols OR technosoil OR technosoils OR anthroposol OR anthroposols OR anthroposoil OR anthroposoils OR anthrosol OR anthrosols OR anthrosoil OR anthrosoils**  37 results screened on title/abstract/document type | 2 |
| The resources centre for ecological engineering of the French Biodiversity Agency (<https://www.genieecologique.fr/> ) | 5/10/22 | Tab “Documentation et Outils”  **Keyword in title:**   - **Technosol: 0 result** - **Sol: 9 results**   screening on title / full text | 1 |
| The Paris Region Institute (<https://www.institutparisregion.fr/> ) | 5/10/22 | Tab “Environnement”, select "Economie circulaire", select “Nos ressources”, select "Publications"  About 7 pages screened: 0 result  Tab “Environnement”, select "Toutes", filter "Publications"  Show all publications, Search  **Keyword “sol”: 0 result** | 0 |
| The French Geological Survey (BRGM) (<https://www.brgm.fr/>) | 7/10/22 | Search **Keyword**   - **Technosol: 0 result**   "Menu", Tab “Résultats et données”, select "Rapports publics"  [Les rapports scientifiques et techniques publics élaborés par le BRGM sont accessibles en ligne, sur le portail InfoTerre du BRGM. Ces rapports sont directement téléchargeables au format PDF. <https://infoterre.brgm.fr/>]  “Rechercher des données”, select in “documents et ouvrages”  **Keyword:**   - **Technosol: 3 results** | 1 |
| The Centre for landscape and urban horticulture (Plante & Cité) (<https://www.plante-et-cite.fr/>) | 7/10/22 | Tab “Ressources” / “Rechercher”  **Keyword:**   - **Technosol: 17 results** | 8 |
| French Centre for Studies and Expertise on Risks, Environment, Mobility and Urban planning (Cerema) <https://www.cerema.fr/fr> (not in the protocol, identified from one reference in Plante & Cite) | 7/10/22 | Tab “Centre de ressources” / “Plateforme documentaire"  **Keyword:**   - **Technosol: 4 results** | 1 |
| **TOTAL** |  |  | **16** |
